# Synergistic Growth Inhibition of HT-29 Colon and MCF-7 Breast Cancer Cells with Simultaneous and Sequential Combinations of Antineoplastics and CNS Drugs

**DOI:** 10.3390/ijms22147408

**Published:** 2021-07-10

**Authors:** Diana Duarte, Armando Cardoso, Nuno Vale

**Affiliations:** 1 OncoPharma Research Group, Center for Health Technology and Services Research (CINTESIS), Rua Doutor Plácido da Costa, 4200-450 Porto, Portugal; dianaduarte29@gmail.com; 2 Faculty of Pharmacy, University of Porto, Rua Jorge Viterbo Ferreira, 228, 4050-313 Porto, Portugal; 3 NeuroGen Research Group, Center for Health Technology and Services Research (CINTESIS), Rua Doutor Plácido da Costa, 4200-450 Porto, Portugal; cardosoa@med.up.pt; 4 Unit of Anatomy, Department of Biomedicine, Faculty of Medicine, University of Porto, Alameda Professor Hernâni Monteiro, 4200-319 Porto, Portugal; 5 Department of Community Medicine, Health Information and Decision (MEDCIDS), Faculty of Medicine, University of Porto, Alameda Professor Hernâni Monteiro, 4200-319 Porto, Portugal

**Keywords:** colorectal cancer, breast cancer, drug synergism, antineoplastic drugs, drug repurposing, CNS drugs, combination therapy

## Abstract

Several central nervous system (CNS) drugs exhibit potent anti-cancer activities. This study aimed to design a novel model of combination that combines different CNS agents and antineoplastic drugs (5-fluorouracil (5-FU) and paclitaxel (PTX)) for colorectal and breast cancer therapy, respectively. Cytotoxic effects of 5-FU and PTX alone and in combination with different CNS agents were evaluated on HT-29 colon and MCF-7 breast cancer cells, respectively. Three antimalarials alone and in combination with 5-FU were also evaluated in HT-29 cells. Different schedules and concentrations in a fixed ratio were added to the cultured cells and incubated for 48 h. Cell viability was evaluated using MTT and SRB assays. Synergism was evaluated using the Chou-Talalay, Bliss Independence and HSA methods. Our results demonstrate that fluphenazine, fluoxetine and benztropine have enhanced anticancer activity when used alone as compared to being used in combination, making them ideal candidates for drug repurposing in colorectal cancer (CRC). Regarding MCF-7 cells, sertraline was the most promising candidate alone for drug repurposing, with the lowest IC_50_ value. For HT-29 cells, the CNS drugs sertraline and thioridazine in simultaneous combination with 5-FU demonstrated the strongest synergism among all combinations. In MCF-7 breast cancer cells, the combination of fluoxetine, fluphenazine and benztropine with PTX resulted in synergism for all concentrations below IC_50_. We also found that the antimalarial artesunate administration prior to 5-FU produces better results in reducing HT-29 cell viability than the inverse drug schedule or the simultaneous combination. These results demonstrate that CNS drugs activity differs between the two selected cell lines, both alone and in combination, and support that some CNS agents may be promising candidates for drug repurposing in these types of cancers. Additionally, these results demonstrate that 5-FU or a combination of PTX with CNS drugs should be further evaluated. These results also demonstrate that antimalarial drugs may also be used as antitumor agents in colorectal cancer, besides breast cancer.

## 1. Introduction

Cancer represents a major health problem worldwide and is the second leading cause of death in the United States of America (USA). In 2021, there were an estimated 1,898,160 new cancer cases and 608,570 cancer deaths in the USA. Colorectal cancer (CRC) represents the second leading cause of death by cancer in the USA, and in 2021, there were an estimated 149,500 newly diagnosed cases and 52,980 deaths caused by this type of cancer. Of these, 17,930 new cases and 3640 deaths occurred in people under the age of 50. Breast cancer represents the second leading cause of death by cancer among women, with an estimated 281,550 new cases and 43,600 deaths in 2021 in the USA [1]. Although surgery and chemotherapy play a major role in the treatment of CRC, the efficacy rate remains very low. The development of new drugs for cancer therapy is, therefore, urgent, but this process is time-consuming, costly and has low approval rates [2]. Additionally, the majority of the new chemotherapeutics have problems related to toxicity, leading to side effects [3]. Thus, it is important to develop and explore novel pharmaceutical strategies to overcome the obstacles associated with the development of new drugs for cancer therapy.

Drug repurposing (or repositioning) and drug combination are two strategies that have gained the attention of many research groups in recent years. Drug repurposing is a strategy that uses drugs that are already approved by the Food and Drug Administration (FDA) in new therapeutic indications besides the original. This strategy presents advantages concerning the development of new drugs, since repurposed drugs are already approved by the FDA and have known safety and toxicity profiles. This allows saving time and money, increasing the likelihood of these drugs entering clinical trials [4].

The drug combination is a strategy that consists of the administration of a cocktail of two or more drugs [5]. This methodology allows overcoming the intratumoral and intertumoral heterogeneity. Intratumoral heterogeneity results from the differential drug response between the different cells of the same tumour, contributing to the progression of the disease and the appearance of drug resistance. The intertumoral heterogeneity corresponds to the heterogeneity between patients with the same type of cancer and makes it difficult to predict the response of different patients to the same therapy [6]. Combination therapies help to overcome these problems and several studies have demonstrated that they are indeed more effective than monotherapy [7,8,9,10,11,12]. The efficacy of the drug combination depends on the schedule of administration (e.g., simultaneous or sequential) and on the design of the combination models [13,14], to make the most of the interaction between the drugs. Pharmacologically, a combination of two or more drugs will be more effective the greater the synergism between the drugs, i.e., the greater the potentiation of its effectiveness compared to the two drugs alone [15].

The repurposing of central nervous system (CNS) drugs has been explored, and several studies demonstrate the effectiveness of this class of drugs in reducing the viability of tumour cells [16,17,18,19,20]. CNS drugs can be divided into three main classes: antipsychotics, antidepressants and anticonvulsants. Antipsychotics and antidepressants can be subdivided according to their mechanism of action in tricyclic antidepressants (TCA), monoamine oxidase inhibitors (MAOI), selective serotonin reuptake inhibitors (SSRI), serotonin and norepinephrine reuptake inhibitors (SNRI), norepinephrine and dopamine reuptake inhibitors (NDRI) and atypical antidepressants [20]. Several CNS drugs have demonstrated potential for drug repurposing, such as imipramine, phenothiazines, trifluoperazine, pimozide and valproic acid. Imipramine, for example, has been studied in different types of cancer, such as glioma [19], breast [21], head and neck carcinoma [22], acute/chronic myeloid leukaemia [23,24], etc. Phenothiazines, a conventional antipsychotic drug family, the members of which work mainly as dopamine D2 antagonists, have also been studied in breast cancer [25], small cell lung carcinoma [18] and oral cancer [26]. Trifluoperazine, an FDA-approved phenothiazine and a D2 receptor antagonist, was already studied in glioblastoma [27] and lung cancer [28], among others. Pimozide, another D2 blocking agent used for Tourette’s Disorder, can fight cancer cells, including the apoptotic effects in cancer cells and the decreased expression of *Bcl-2* [29]. Valproate (Valproic acid) is an anti-epileptic drug that blocks Na^+^ channels, GABA transaminase and Ca^2+^ channels. This drug is used in epilepsy, migraine seizures and acute manic episodes. Several studies suggest its beneficial role in fighting lymphoma [30], prostate [31] and breast cancer [32] and bladder [33] and hepatocellular carcinoma [34], among others.

**Scheme 1:**
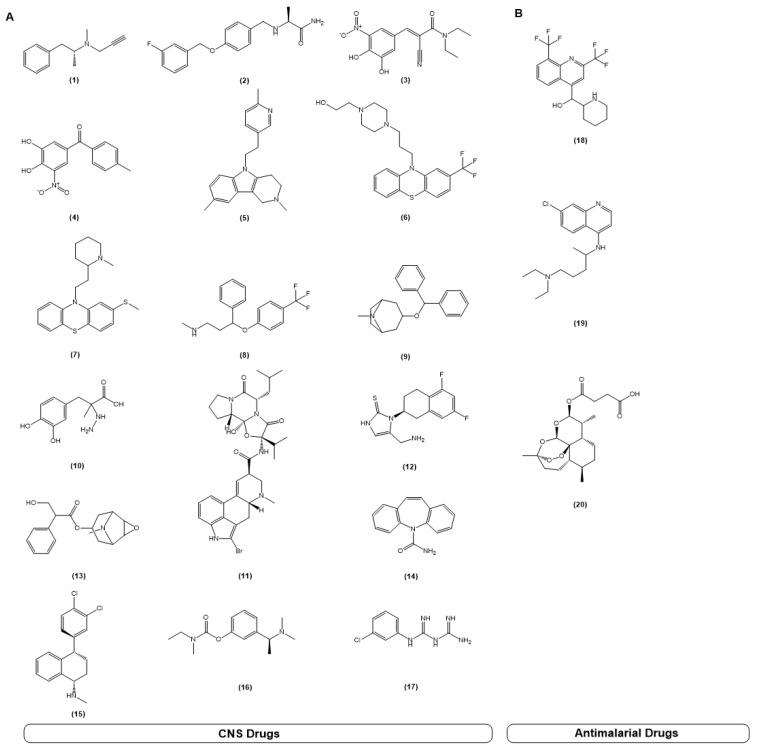
Chemical structures of the drugs used in combination. (**A**) CNS drugs: (**1**) selegiline, (**2**) safinamide, (**3**) entacapone, (**4**) tolcapone, (**5**) latrepirdine, (**6**) fluphenazine, (**7**) thioridazine, (**8**) fluoxetine, (**9**) benztropine, (**10**) carbidopa, (**11**) bromocriptine, (**12**) nepicastat, (**13**) scopolamine, (**14**) carbamazepine, (**15**) sertraline and (**16**) rivastigmine. (**B**) Antimalarial drugs: (**18**) mefloquine, (**19**) chloroquine and (**20**) artesunate.

In this work, we hypothesised that different CNS agents (Scheme 1A and Table 1) could synergistically act with 5-fluorouracil (5-FU) and paclitaxel (PTX) in the CRC and breast cancer treatments, respectively. 5-FU is an antineoplastic drug commonly used in CRC therapy, but its use has several limitations, including its short half-life, high cytotoxicity, and low bioavailability [35]. PTX is a chemotherapeutic agent that is a mitotic inhibitor, used for the treatment of advanced carcinoma of the ovary, and other various cancers including breast and lung cancer. PTX use is limited by the appearance of drug resistance and its side effects [36]. This combination model consists of the combination of an antineoplastic drug and different repurposed drugs, and aims to improve the activity of the reference drug and simultaneously reduce its therapeutic dose, by using drugs with acceptable toxicological profiles.

Recently, our group also developed a new combination model using different antimalarials and antineoplastic drugs in MCF-7 breast cancer cells [37]. Several antimalarials have been combined with doxorubicin and paclitaxel, two antineoplastic agents commonly used in breast cancer therapy. The results were very promising, and it was found that the best combinations corresponded to the antimalarials mefloquine, chloroquine and artesunate [37]. Although the relationship between (familial) breast cancer and colorectal cancer is a controversial subject, recently, it was discovered that rare mutations in the *NTHL1* gene, which was originally associated with CRC, also cause breast cancer [38]. For this reason, we decided to also include these antimalarials (Scheme 1B) in this study to confirm the anti-tumoral activity of this class of drugs in the HT-29 colon cancer cells. We have demonstrated that the combination of 5-FU and some antimalarials also induces anti-tumour effects in HT-29 cells. Interestingly, for artesunate, we discovered that the drug schedule influences the anticancer effect of this combination, being greater when artesunate is given before 5-FU to HT-29 cells.

We demonstrate that some CNS drugs such as fluphenazine, fluoxetine and benztropine work better alone than in combination with 5-FU to reduce the viability of HT-29 colon cancer cells. In MCF-7 cells, sertraline was the most promising repurposed drug when used alone, with the lowest IC_50_ value. Compared to HT-29 cells, the IC_50_ obtained for the tested CNS drugs was higher in breast cancer cells, demonstrating a better efficacy of these drugs in CRC. We also found that the combination of 5-FU with sertraline and thioridazine induces a greater anti-tumour effect compared to each drug alone in these cells. In combination, results for MCF-7 were more promising than in HT-29 cells, with the combinations of PTX with fluoxetine, benztropine and fluphenazine resulting in a higher number of synergistic pairs.

## 2. Results

### 2.1. HT-29 Colorectal Cancer Cells

#### 2.1.1. The Effect of 5-FU as the Single Agent on Cellular Viability

**Figure 1:**
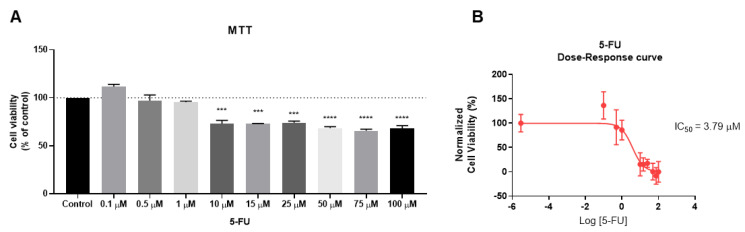
The effect of 5-FU on HT-29 cells. (**A**) Cell viability and (**B**) dose-response. Cells were cultured in the presence of increasing concentrations of 5-FU, and after 48 h, the MTT assay was performed to measure the cellular viability. Values are expressed in percentage of control and represent means ± SEM. Each experiment was done three times independently (*n* = 3) *** statistically significant vs. control at *p* < 0.001. **** statistically significant vs. control at *p* < 0.0001.

We analysed the anti-tumour potential of the antineoplastic drug 5-FU in the HT-29 colorectal cancer cell line, to confirm its efficacy in this type of cancer. Cells were treated with 5-FU in concentrations ranging 0.1–100 μM for 48 h and cell survival was evaluated by MTT, a viability assay that measures mitochondrial activity. The results of the MTT assay for 5-FU are given in Figure 1A. Based on these results, a dose-response curve was obtained and the IC_50_ value for 5-FU was calculated (Figure 1B). This value was further used in the combinations. Our results revealed a significant activity of 5-FU at concentrations above 10 µM, with little differences in cell viability among the higher concentrations. The cells displayed a mild response to the cytotoxic effect of 5-FU, with less than 4 µM killing almost 50% of cells. These results support the anti-cancer activity of 5-FU in the treatment of CRC and justify its use in the combinations proposed in this study.

#### 2.1.2. The Effect of CNS Drugs and Antimalarial Drugs as Single Agents on Cellular Viability

We next evaluated the probable antitumor effect of different CNS drugs as single agents, namely selegiline, entacapone, tolcapone, latrepirdine, fluphenazine, safinamide, carbidopa, scopolamine, benztropine, thioridazine, fluoxetine, nepicastat and bromocriptine in HT-29 colon cancer cells. In this study, we have also included three antimalarial drugs (mefloquine, chloroquine and artesunate) based on our previous results [37], to confirm if these drugs would maintain their anti-cancer activity in another cell line besides the MCF-7 breast cancer cells. HT-29 cells were treated with increasing concentrations of each drug, starting from 1 µM to 100 µM to evaluate cell viability after 48 h of treatment.

**Figure 2:**
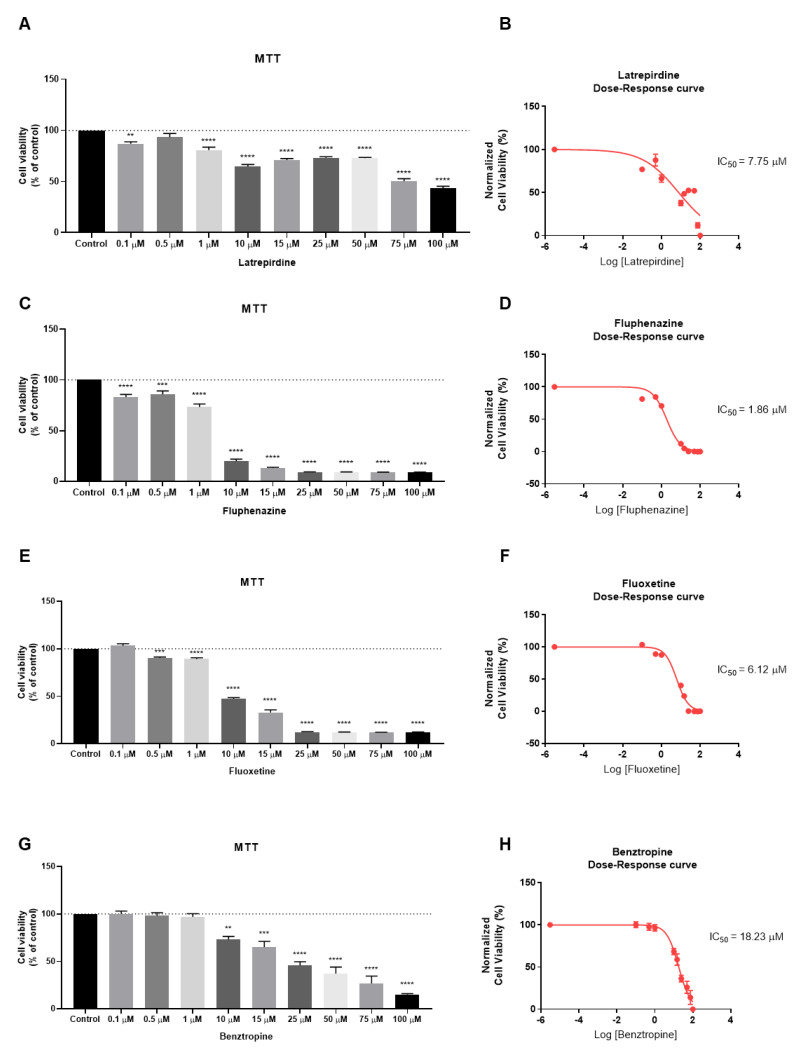
The effects of some CNS drugs on HT-29 cells. (**A**) The effect of latrepirdine on cell viability and (**B**) the dose-response curve. (**C**) The effect of fluphenazine on cell viability and (**D**) the dose-response curve. (**E**) The effect of fluoxetine on cell viability and (**F**) the dose-response curve. (**G**) The effect of benztropine on cell viability and (**H**) the dose-response curve. Cells were cultured in the presence of increasing concentrations of each drug, and after 48 h, the MTT assay was performed to measure the cellular viability. Values are expressed in percentage of control and represent means ± SEM. Each experiment was done three times independently (*n* = 3); ** statistically significant vs. control at *p* < 0.01. *** statistically significant vs. control at *p* < 0.001. **** statistically significant vs. control at *p* < 0.0001.

**Figure 3:**
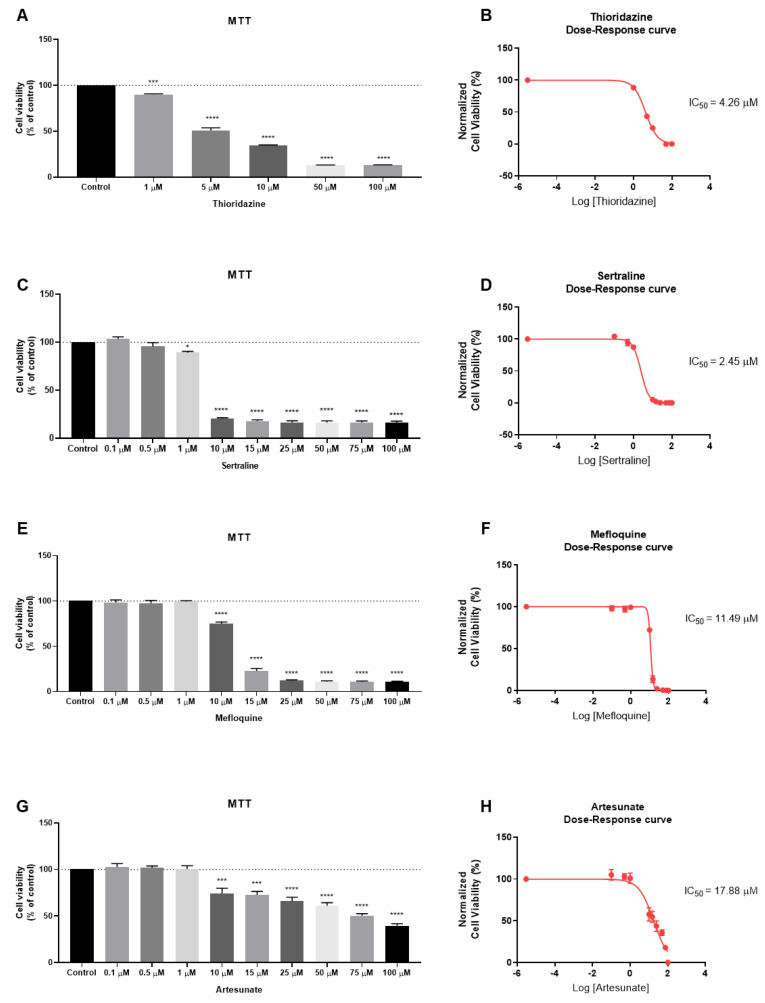
The effects of some CNS drugs and antimalarial drugs on HT-29 cells. (**A**) The effect of thioridazine on cell viability and (**B**) the dose-response curve. (**C**) The effect of sertraline on cell viability and (**D**) the dose-response curve. (**E**) The effect of mefloquine on cell viability and (**F**) the dose-response curve. (**G**) The effect of artesunate on cell viability and (**H**) the dose-response curve. Cells were cultured in the presence of increasing concentrations of each drug, and after 48 h, the MTT assay was performed to measure the cellular viability. Values are expressed in percentage of control and represent means ± SEM. Each experiment was done three times independently (*n* = 3); * statistically significant vs. control at *p* < 0.05. *** statistically significant vs. control at *p* < 0.001. **** statistically significant vs. control at *p* < 0.0001.

Based on the MTT results, we found that latrepirdine, fluphenazine, fluoxetine, benztropine, thioridazine, sertraline, mefloquine and artesunate displayed significant anti-tumour activity in HT-29 cells. Cytotoxic effects of latrepirdine (Figure 2A) were significant even in concentrations of 1 µM, with 7.75 µM causing a reduction of more than 50% of the cells (Figure 2B). Fluphenazine anti-tumour effect was the strongest among all drugs tested alone and concentrations above 10 µM killed almost all cells (Figure 2C). Indeed, the IC_50_ obtained for fluphenazine was the lowest and it was less than 2 µM (Figure 2D). The MTT assay for fluoxetine treatment demonstrated a strong cytotoxic effect of this CNS drug in HT-29 cells for all concentrations tested above 10 µM (Figure 2E). The dose-response curve for fluoxetine revealed an IC_50_ value of 6.12 µM (Figure 2F). Benztropine showed significant anti-tumour effects in concentrations above 10 µM (Figure 2G) and the IC_50_ value obtained was 18.23 µM. Thioridazine treatment also significantly decreased HT-29 cell viability, from 1 µM to 100 µM, with strong effects for all concentrations above 5 µM (Figure 3A) and an IC_50_ value of 4.26 µM (Figure 3B). Treatment with sertraline at doses above 1 µM for 48 h had a strong effect on the cell viability (Figure 3C), resulting in an IC_50_ value of less than 3 µM (Figure 3D). All concentrations of the antimalarial drug mefloquine above 10 µM showed a strong effect on the cell viability of HT-29 cells, with more than 50% of the cells being not viable (Figure 3E). The dose-response curve resulted in a value of 11.49 µM for the IC_50_ (Figure 3F). Artesunate, another antimalarial drug, also demonstrates good efficacy against these cells in all concentrations above 10 µM (Figure 3G) and an IC_50_ value under 20 µM (Figure 3H). MTT assays for the others CNS drugs and chloroquine demonstrate a lack of efficacy of these drugs on the reduction of HT-29 cell viability or IC_50_ above 20 µM and were discarded from the drug combinations. These results demonstrate that both CNS agents and antimalarial drugs are good candidates for use in combination with 5-FU. Table 2 summarises the IC_50_ obtained for all drugs tested alone in this work.

#### 2.1.3. The Effect of Various Combinations of 5-FU and Different CNS Agents and Antimalarial Drugs

After finding the best candidates for drug repurposing in CRC therapy and their IC_50_ value, we evaluated the combination of 5-FU with each drug using the model of combination developed in our previous work [37]. Specifically, HT-29 cells were treated with the two drugs alone or combined in a fixed ratio, in the concentrations of 0.25 × IC_50_, 0.5 × IC_50_, IC_50_, 2 × IC_50_ and 4 × IC_50_, and two cell-based assays were performed: MTT and SRB. Morphological evaluation of cells treated with each drug alone and in combination was also done. The most promising drugs for drug combination were selected according to their IC_50_ value. To do so, the combination of 5-FU and each drug of Table 2 with an IC_50_ value under 20 µM was evaluated: latrepirdine, fluphenazine, fluoxetine, benztropine, thioridazine, sertraline, mefloquine and artesunate.

**Figure 4:**
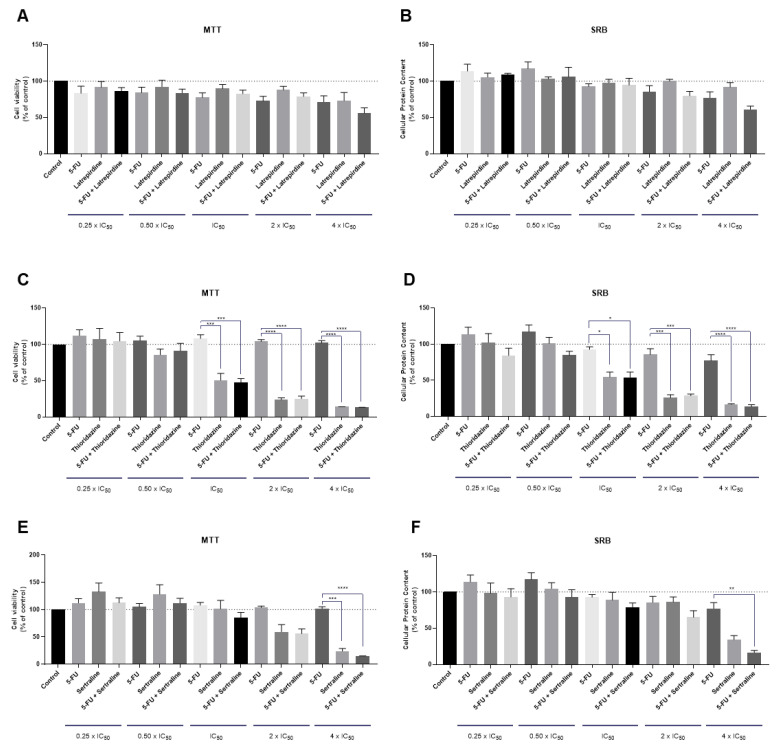

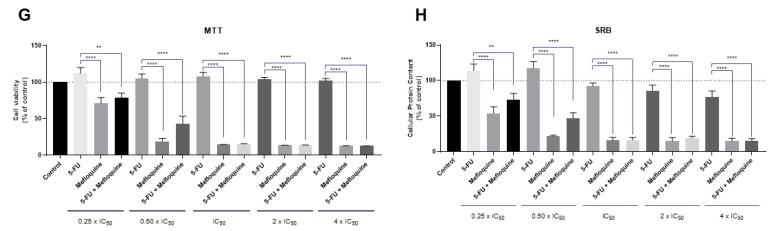
Growth inhibition of HT-29 after 48 h of combination therapy, by MTT (left) and SRB assays (right). Cells were exposed to concentrations of each drug of 0.25, 0.5, 1, 2 and 4 times their IC_50_ and the cell viability was evaluated by MTT and SRB assays. The drugs in combination were co-administered at the same time. (**A**) The effect of 5-FU plus latrepirdine on cell viability and (**B**) cell protein synthesis. (**C**) The effect of 5-FU plus thioridazine on cell viability and (**D**) cell protein synthesis. (**E**) The effect of 5-FU plus sertraline on cell viability and (**F**) cell protein synthesis. (**G**) The effect of 5-FU plus mefloquine on cell viability and (**H**) cell protein synthesis. Values are expressed in percentage of control and represent means ± SEM. Each experiment was done three times independently (*n* = 3); * statistically significant vs. control at *p* < 0.05. ** statistically significant vs. control at *p* < 0.01. *** statistically significant vs. control at *p* < 0.001. **** statistically significant vs. control at *p* < 0.0001.

**Figure 5:**
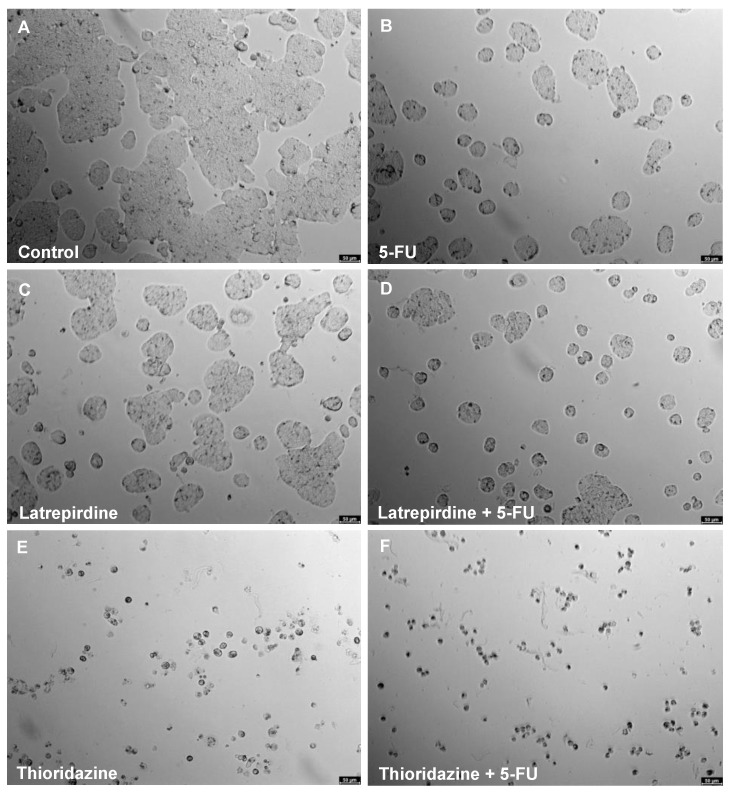

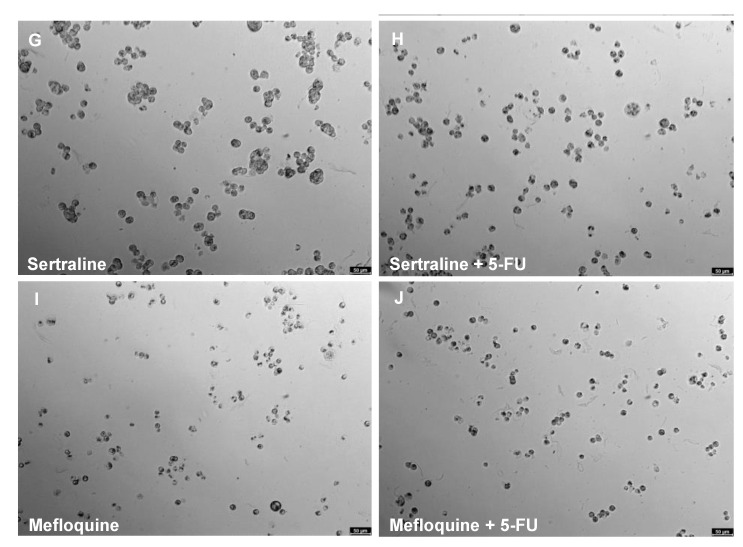
Microscopic cellular visualisation of HT-29 cells after 48 h of incubation with vehicle (**A**), 5-FU (**B**), latrepirdine (**C**), latrepirdine + 5-FU (**D**), thioridazine (**E**), thioridazine + 5-FU (**F**), sertraline (**G**), sertraline + 5-FU (**H**), mefloquine (**I**) and mefloquine + 5-FU (**J**) at concentrations of 4 × IC_50_ of each drug.

**Figure 6:**
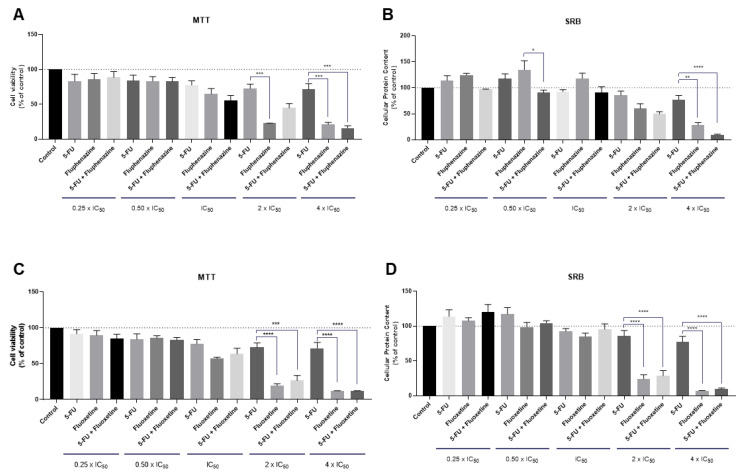

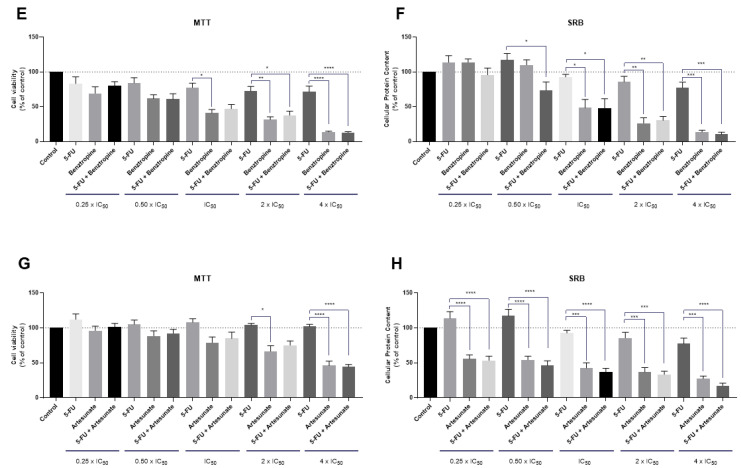
Growth inhibition of HT-29 after 48 h of combination therapy, by MTT (left) and SRB assays (right). Cells were exposed to concentrations of each drug of 0.25, 0.5, 1, 2 and 4 times their IC_50_ and cell viability were evaluated by MTT and SRB assays. The drugs in combination were co-administered at the same time. (**A**) The effect of 5-FU plus fluphenazine on cell viability and (**B**) cell protein synthesis. (**C**) The effect of 5-FU plus fluoxetine on cell viability and (**D**) cell protein synthesis. (**E**) The effect of 5-FU plus benztropine on cell viability and (**F**) cell protein synthesis. (**G**) The effect of 5-FU plus artesunate on cell viability and (**H**) cell protein synthesis. Values are expressed in percentage of control and represent means ± SEM. Each experiment was done three times independently (*n* = 3); * statistically significant vs. control at *p* < 0.05. ** statistically significant vs. control at *p* < 0.01. *** statistically significant vs. control at *p* < 0.001. **** statistically significant vs. control at *p* < 0.0001.

**Figure 7:**
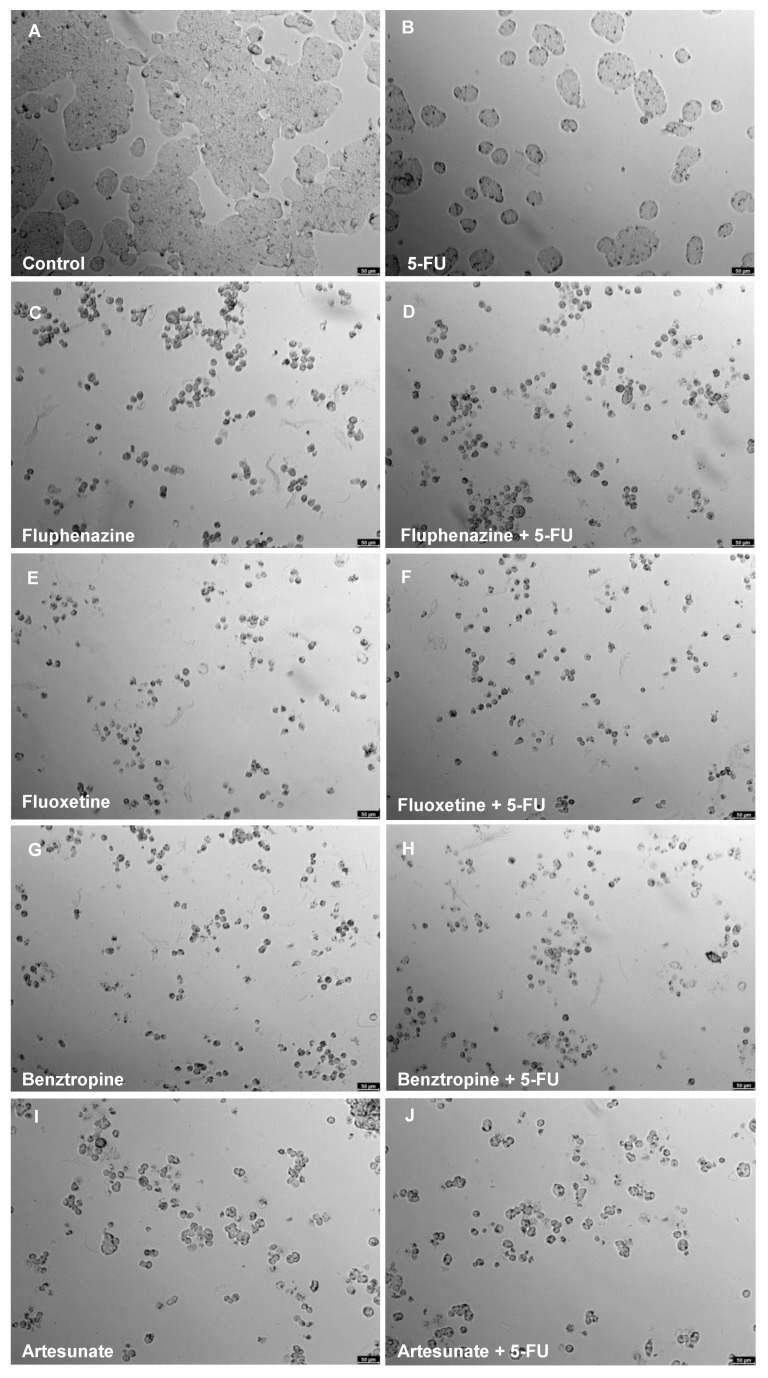
Microscopic cellular visualisation of HT-29 cells after 48 h of incubation with vehicle (**A**), 5-FU (**B**), fluphenazine (**C**), fluphenazine + 5-FU (**D**), fluoxetine (**E**), fluoxetine + 5-FU (**F**), benztropine (**G**), benztropine + 5-FU (**H**), artesunate (**I**) and artesunate + 5-FU (**J**) at concentrations of 4 × IC_50_ of each drug.

When combined with 5-FU, latrepirdine did not have any significant anti-cancer effects, at any concentration, both by MTT and SRB assay (Figure 4A,B, respectively). The combination with thioridazine resulted in a similar reduction of cell viability and cell protein synthesis as thioridazine alone, at concentrations higher than IC_50_. At a concentration of 4 × IC_50_, the combination of 5-FU plus thioridazine demonstrated enhanced but not significant anticancer effects than thioridazine (Figure 4C,D). The combination with 5-FU and sertraline also demonstrated significant anticancer effects compared to 5-FU alone, at a higher concentration, for both assays (Figure 4E,F). A small difference between sertraline and sertraline+5-FU is seen at the concentration of 4 × IC_50_, but this is not significant. The combination with mefloquine resulted in all concentrations showing a greater anticancer effect than 5-FU alone (Figure 4G,H). The activity seen on these combinations can be the result of the strong anticancer activity of mefloquine alone. Morphologically, the results are in agreement with the MTT and SRB assays. At concentrations of 4 × IC_50_, all combinations resulted in a decrease of cell number and smaller and rounder cells, comparing with control cells and 5-FU, which is indicative of cell death (Figure 5). In the combinations 5-FU plus fluphenazine, fluoxetine, benztropine and artesunate, we found out that for a concentration of 2 × IC_50_, the results of the combined drugs were worse than the repurposed drugs alone, demonstrating a kind of competition mechanism between the two drugs when administered together, mainly in the MTT assays. Additionally, for the higher concentrations (4 × IC_50_), the results obtained for the combined drugs did not show improvements concerning the repurposed drugs alone (Figure 6). Microscopically, at concentrations of 4 × IC_50_, differences between cells were only found between 5-FU, control cells and treated cells; differences between single drugs and drug combinations were very subtle and both treatments resulted in the decreasing of cell number, less aggregate formation and rounded cells (Figure 7).

#### 2.1.4. Synergistic Combinations of 5-FU and CNS Agents/Antimalarial Drugs

**Figure 8:**
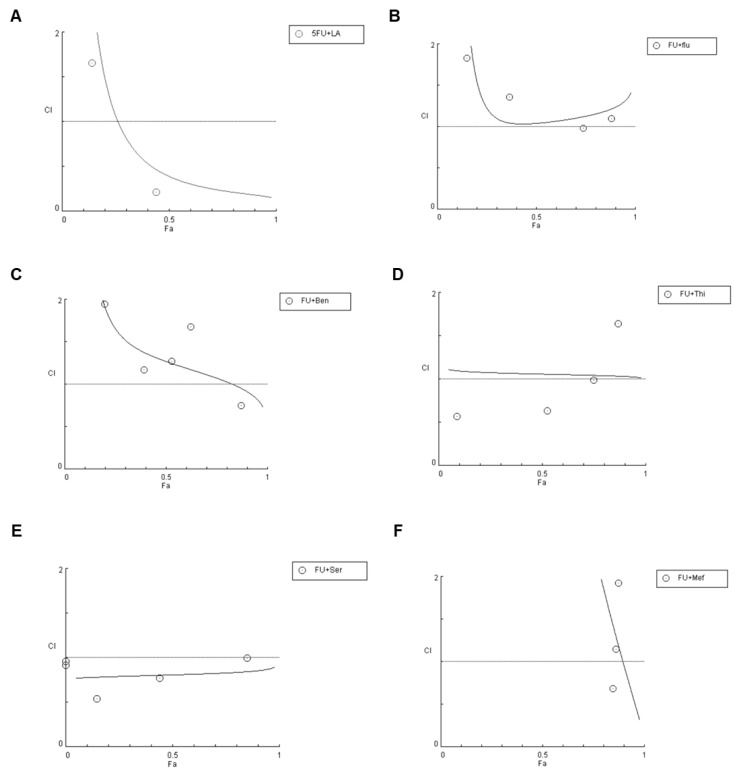

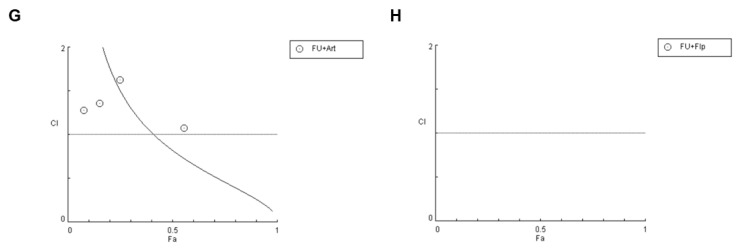
Chou-Talalay method Fa-CI plot of 5-FU plus latrepirdine (**A**), fluoxetine (**B**), benztropine (**C**), thioridazine (**D**), sertraline (**E**), mefloquine (**F**), artesunate (**G**) and fluphenazine (**H**). CI was plotted on the *y*-axis as a function of effect level (Fa) on the *x*-axis to evaluate drug synergism. CI < 1, CI = 1 and CI > 1 refers to synergism, additivity and antagonism, respectively.

To investigate the effects of the combinations of 5-FU with the previous drugs, and after finding the most promising ones based on MTT and SRB assays, the combination index (CI) was calculated using the Chou-Talalay method, using the CompuSyn software. CI was plotted on the *y*-axis as a function of effect level (Fa) on the *x*-axis to assess drug synergism. The fractional effect is a value between 0 and 1, where 0 means that the drug did not affect cell viability and 1 means that the drug produced a full effect on decreasing cell viability. A combination of 5-FU plus latrepirdine demonstrated little synergism with only one synergic pair (Figure 8A), with an Fa value of 0.44 (Table 3). Both combinations of 5-FU plus fluoxetine and benztropine demonstrated synergism just for one pair (Figure 8B,C, respectively), with Fa values of 0.73 and 0.87, respectively (Table 3). The combination with thioridazine was one of the most promising ones, with three synergic pairs (Figure 8D) and a Fa value reaching 0.75 (Table 3). For sertraline, all combinations were synergic (Figure 8E) and produced a Fa value of 0.85 (Table 3). The combination of 5-FU and mefloquine also resulted in one synergic pair (Figure 8F), with an Fa value of 0.848 (Table 3). A combination of artesunate and fluphenazine with 5-FU did not result in any synergism (Figure 8G,H, respectively), with CI > 1 for all pairs of concentrations (Table 3). Together, these results demonstrate that some CNS agents, such as sertraline and thioridazine, may be promising to evaluate future combinations.

Besides the Chou-Talalay method, drug interactions were also evaluated by the Bliss Independence and Highest Single Agent (HSA) methods, using the SynergyFinder 2.0 software. This software is a web application for interactive analysis and visualisation of multi-drug combination profiling data by different synergism evaluation methods. The Bliss independence model assumes a stochastic process in which two drugs produce their effects independently, and the expected combination effect can be calculated based on the probability of independent events [57]. The HSA model is one of the simplest reference models for synergism evaluation and states that the expected combination effect is the maximum of the single drug responses at corresponding concentrations. In this software, the synergy score for a drug combination is averaged over all the dose combination measurements, giving a positive or negative value, corresponding to synergism or antagonism, respectively. The 2D and 3D synergy maps highlight synergistic and antagonistic dose regions in red and green colours, respectively [57].

**Figure 9:**
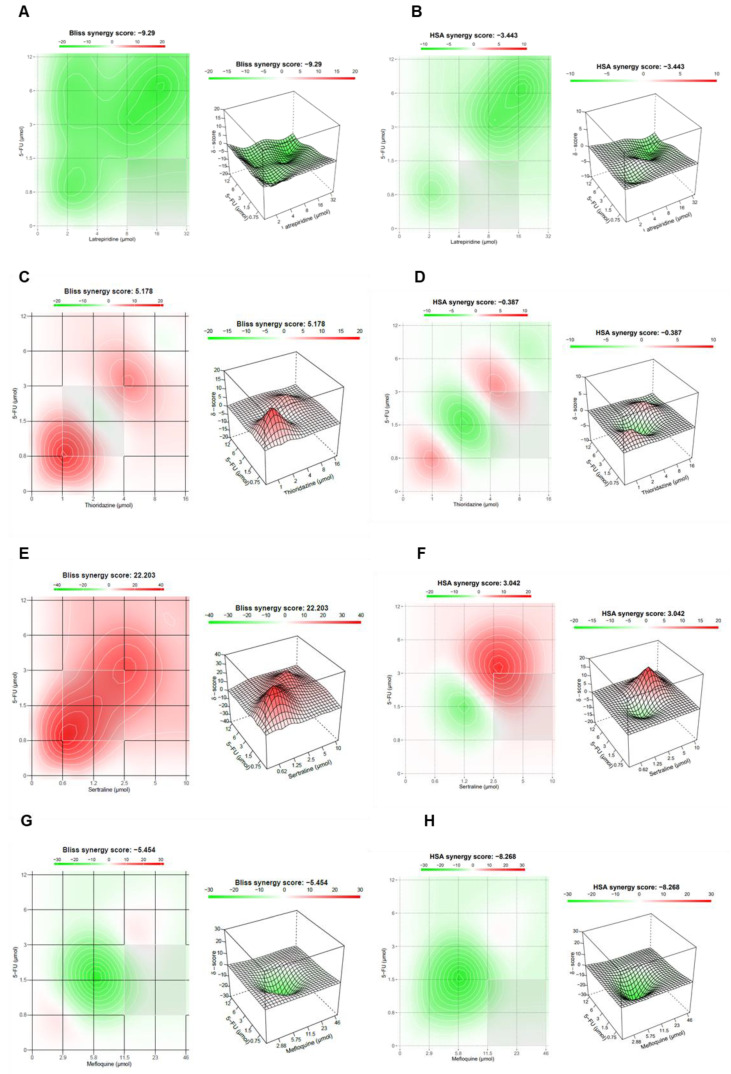
Bliss (**left**) and HAS (**right**) synergy plots of 5-FU plus latrepirdine (**A**,**B**), thioridazine (**C**,**D**), sertraline (**E**,**F**) and mefloquine (**G**,**H**).

**Figure 10:**
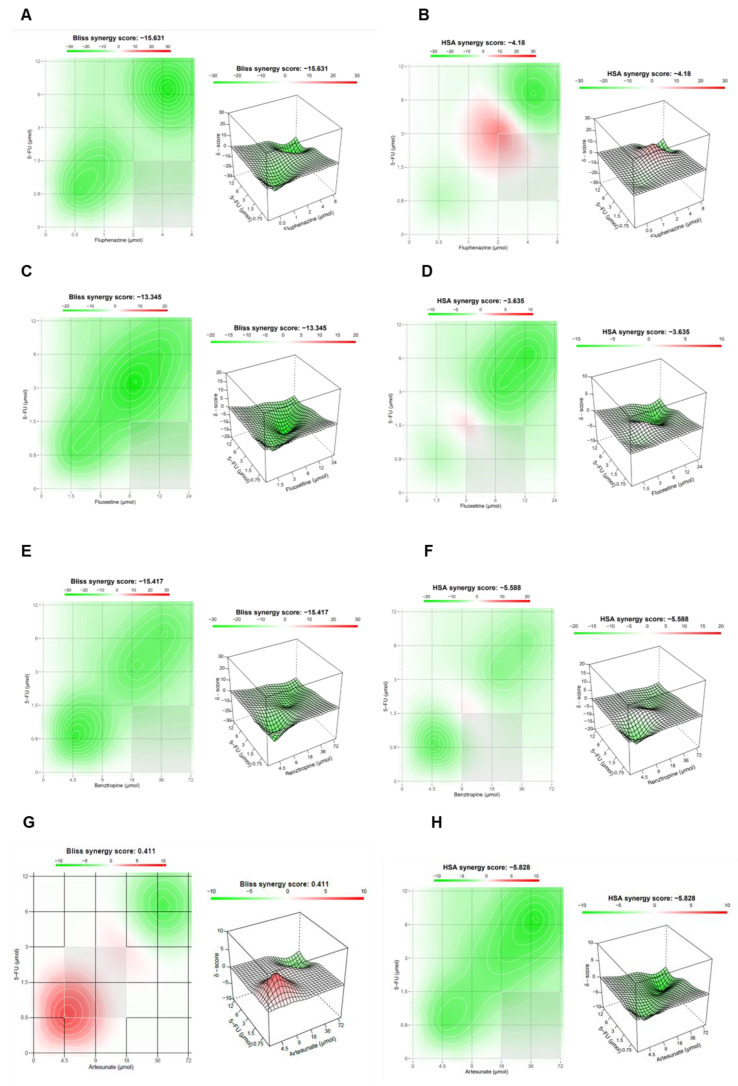
Bliss (**left**) and HAS (**right**) synergy plots of 5-FU plus fluphenazine (**A**,**B**), fluoxetine (**C**,**D**), benztropine (**E**,**F**) and artesunate (**G**,**H**).

Latrepirdine in combination with 5-FU, both by Bliss and HSA models (Figure 9A,B, respectively), demonstrated a negative synergy score, in line with the Chou-Talalay results, indicating antagonism for all pairs. Thioridazine demonstrated synergism by the Bliss model, with a positive synergy score of 5.178 (Figure 9C). The results for the HSA model demonstrated antagonism, but some regions of synergy, as represented in red in Figure 9D. In line with the previous results, the combination of 5-FU with sertraline resulted in strong synergism, both in the Bliss (Figure 9E) and HSA models (Figure 9F), with synergy scores of 22.203 and 3.042, respectively. For mefloquine, no synergism was observed using the Bliss and HSA models (Figure 9G,H, respectively). By the Bliss model, fluphenazine combined with 5-FU resulted in a negative synergy score, demonstrating antagonism (Figure 10A). By the HSA models, the general synergy score was also negative but with a region in the 2D/3D plot demonstrating a pair of concentrations with synergic behaviour (Figure 10B). Fluoxetine and benztropine did not show any synergism in Bliss and HSA models, demonstrating an antagonistic behaviour between these drugs and 5-FU (Figure 10C–F). Contrary to the previous results obtained by the Chou-Talalay method, the combination of 5-FU plus artesunate as evaluated by the Bliss Method resulted in a positive synergy score of 0.411, with a red region on the 2D/3D plot in the lowest concentrations (Figure 10G). Using the HSA model, the synergy score was negative, demonstrating antagonism (Figure 10H). These results demonstrate that the choice of synergy evaluation model can give slightly different results regarding the synergy evaluation of drug combinations, although these reference models produce similar results most of the time.

#### 2.1.5. The Effect of Different Combination Schedules of 5-FU and Different CNS Agents and Antimalarial Drugs

**Figure 11:**
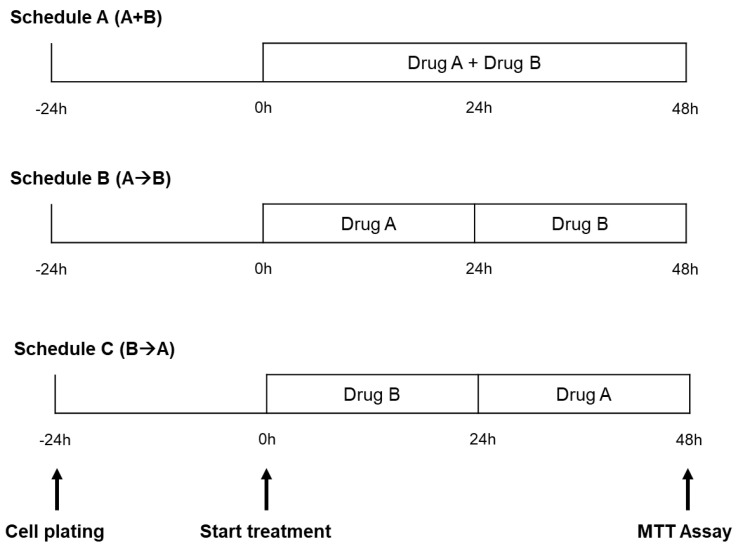
The three combination schedules designed for evaluating 5-FU combination with fluphenazine, fluoxetine, benztropine and artesunate. Schedule A represents cells treated concomitantly with 5-FU and each repurposed drug for 48 h. Schedule B represents cells pre-treated with 5-FU for 24 h followed by each repurposed drug for another 24 h. For schedule C, cells were pre-treated with each repurposed drug for 24 h, followed by 5-FU for another 24 h.

**Figure 12:**
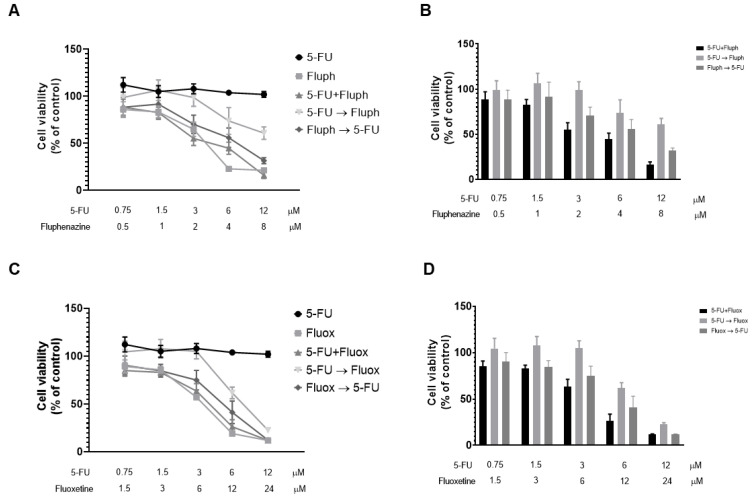

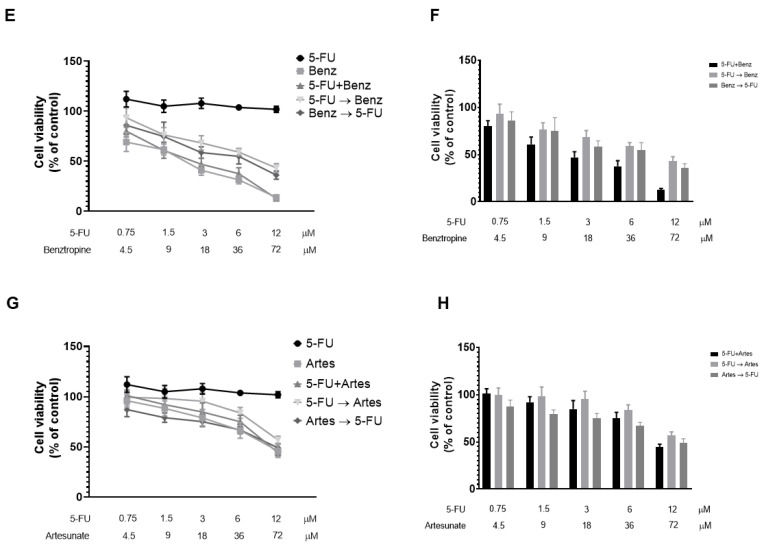
HT-29 cells were exposed to sequential 5-FU and (**A**,**B**) fluphenazine (**C**,**D**) fluoxetine (**E**,**F**) benztropine and (**G**,**H**) artesunate using constant ratios of the IC_50_ dose. Values are expressed in percentage of control and represent means ± SEM. Each experiment was done three times independently (*n* = 3).

Based on the MTT assay results (Figure 6), the combination of 5-FU with fluphenazine, fluoxetine, benztropine and artesunate seem to demonstrate some kind of competition between the two drugs, with the results for the combination being worse than for the repurposed drugs alone. We design a new model of combination for these pairs of drugs and evaluated the influence of the drug schedule on HT-29. We hypothesise that if we administered the drugs at different times (sequential), the results would be better, due to non-competition between the two drugs. To do so, we tested three schedules (Figure 11): simultaneous administration (Schedule A), drug A prior drug B (Schedule B) and drug B prior drug A (Schedule C). We found out that for all CNS drugs, simultaneous administration produced better results in reducing cell viability than other schedules (Figure 12A–F). Interestingly, we found that all CNS drugs alone demonstrated better antitumor activity than in combination, indicating these drugs are ideal candidates for drug repurposing. For artesunate, we found out that the administration of artesunate prior to 5-FU produced better results than other drug schedules (Figure 12G,H).

#### 2.1.6. Synergism Evaluation of Different Combination Schedules of 5-FU and CNS Agents/Antimalarial Drugs

**Figure 13:**
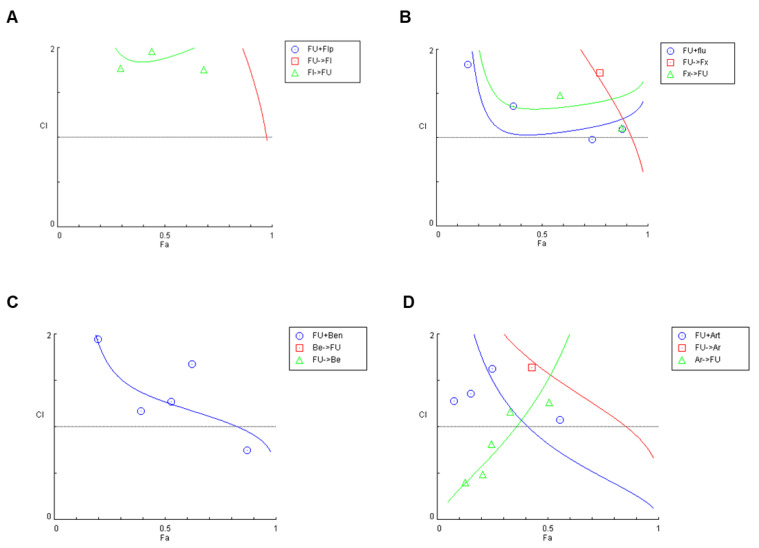
Chou-Talalay method Fa-CI plot of three schedule-dependent combinations of 5-FU plus fluphenazine (**A**), fluoxetine (**B**), benztropine (**C**) and artesunate (**D**). CI was plotted on the *y*-axis as a function of effect level (Fa) on the *x*-axis to evaluate drug synergism. CI < 1, CI = 1 and CI > 1 refers to synergism, additivity and antagonism, respectively.

Based on the previous results, we also analysed the drug interactions in these combinations to evaluate if there were differences in the CI values between the three schedules of administration. For fluphenazine, there were no differences between the simultaneous administration and the sequential administration of the drugs, and all pairs were antagonists (Figure 13A). For fluoxetine, only one pair in the simultaneous administration was synergic, and in sequential administration, no synergism could be seen, so the simultaneous combination seems to be advantageous over the sequential (Figure 13B). The same was observed for benztropine, demonstrating a lack of efficacy in sequential administration (Figure 13C). Contrary to these drugs, a combination with artesunate in sequential form, with artesunate being given prior to 5-FU, seems to have better results compared to 5-FU prior artesunate and simultaneous administration, resulting in three synergistic pairs (CI < 1) (Figure 13D). Table 4 shows the CI values obtained for each combination, depending on the drug schedule.

### 2.2. MCF-7 Breast Cancer Cells

#### 2.2.1. The Effect of CNS Drugs as Single Agents on Cellular Viability

Finally, we evaluated the cytotoxic effect of the most promising CNS drugs in MCF-7 breast cancer cells, both alone and in combination. This time, we combined these drugs with paclitaxel (PTX), an antineoplastic drug that is used for the treatment of breast cancer instead of 5-FU, as previous results from our group revealed that this drug is not very effective against MCF-7 breast cancer cells. Based on HT-29 results, we selected thioridazine, benztropine, fluoxetine, fluphenazine, sertraline and latrepirdine and evaluated their effect on MCF-7 viability. As previous, MCF-7 cells were treated with increasing concentrations of each repurposed drug, starting from 1 µM to 100 µM to evaluate cell viability after 48 h treatment.

**Figure 14:**
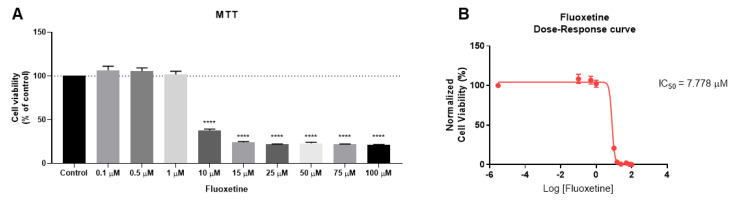

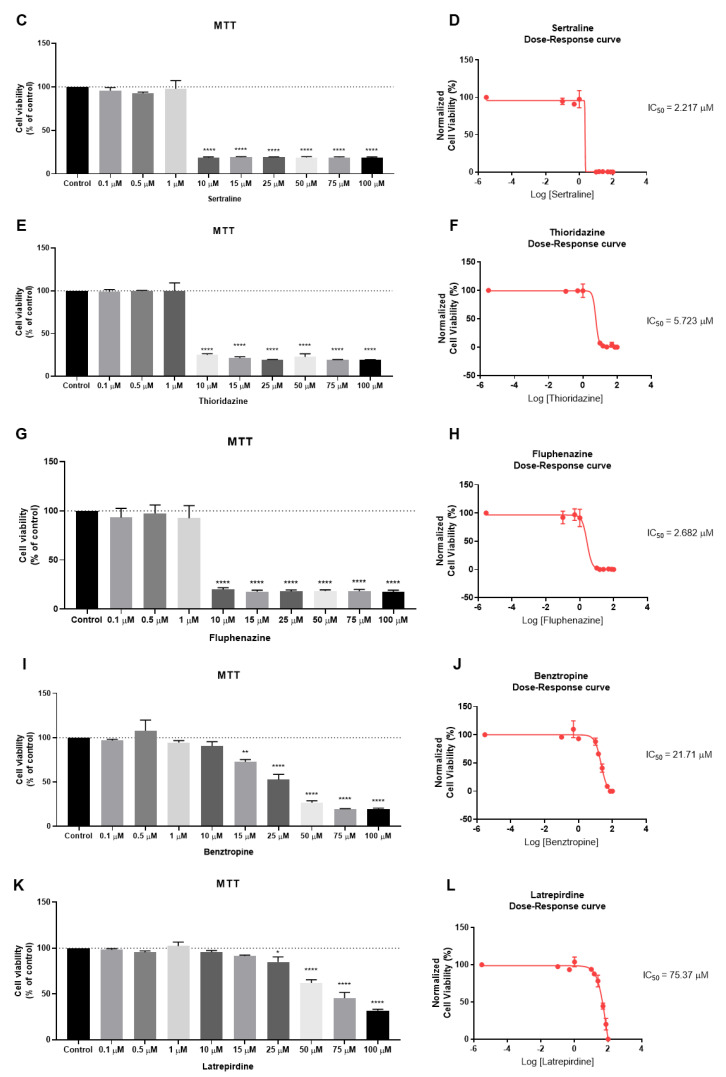
The effects of some CNS drugs on MCF-7 cells. (**A**) The effect of fluoxetine on cell viability and (**B**) the dose-response curve. (**C**) The effect of sertraline on cell viability and (**D**) dose-response curve. (**E**) The effect of thioridazine on cell viability and (**F**) the dose-response curve. (**G**) The effect of fluphenazine on cell viability and (**H**) the dose-response curve. (**I**) The effect of benztropine on cell viability and (**J**) the dose-response curve. (**K**) The effect of latrepirdine on cell viability and (**L**) the dose-response curve. Cells were cultured in the presence of increasing concentrations of each drug, and after 48 h, the MTT assay was performed to measure the cellular viability. Values are expressed in percentage of control and represent means ± SEM. Each experiment was done three times independently (*n* = 3); * statistically significant vs. control at *p* < 0.05. ** statistically significant vs. control at *p* < 0.01. **** statistically significant vs. control at *p* < 0.0001.

Based on the MTT results, we found that all tested CNS drugs displayed significant anti-tumour activity in MCF-7 cells. Cytotoxic effects of fluoxetine (Figure 14A) were significant in concentrations above 10 µM, with 7.78 µM causing a reduction of more than 50% of the cells (Figure 14B). The anti-tumour effect of sertraline was the strongest among all drugs tested alone, and concentrations above 10 µM killed almost all cells (Figure 14C). The IC_50_ value obtained for sertraline was the lowest, i.e., about 2.22 µM (Figure 14D). MTT results for thioridazine demonstrated a strong cytotoxic effect of this CNS drug in HT-29 cells for all concentrations tested above 10 µM (Figure 14E). The dose-response curve for thioridazine resulted in an IC_50_ value of 5.72 µM (Figure 14F). Fluphenazine showed significant anti-tumour effects similar to sertraline in concentrations above 10 µM (Figure 14G) and the IC_50_ value obtained was 2.68 µM (Figure 14H). Benztropine and latrepirdine effects on MCF-7 viability were the worst among all drugs tested. Benztropine treatment significantly decreased MCF-7 breast cancer cell viability for all concentrations above 15 µM (Figure 14I) and an IC_50_ value of 21.71 µM (Figure 14J). Only treatments with latrepirdine at doses above 25 µM for 48 h had a significant effect on the cell viability (Figure 14K), resulting in an IC_50_ value of more than 70 µM (Figure 14L).

These results demonstrate that CNS agents, such as fluoxetine, sertraline, benztropine, fluphenazine and thioridazine, are good candidates to be used in combination with PTX. Table 5 shows a comparison between the IC_50_ obtained for these drugs in the two cell lines (MCF-7 and HT-29). Compared to the previous results, it is possible to verify that all IC_50_ values obtained for MCF-7 breast cancer cells were higher than the ones obtained for HT-29 colon cancer cells, except for sertraline, demonstrating that these drugs alone have less potency in breast cancer cells.

#### 2.2.2. The Effect of Various Combinations of PTX and Different CNS Agents

**Figure 15:**
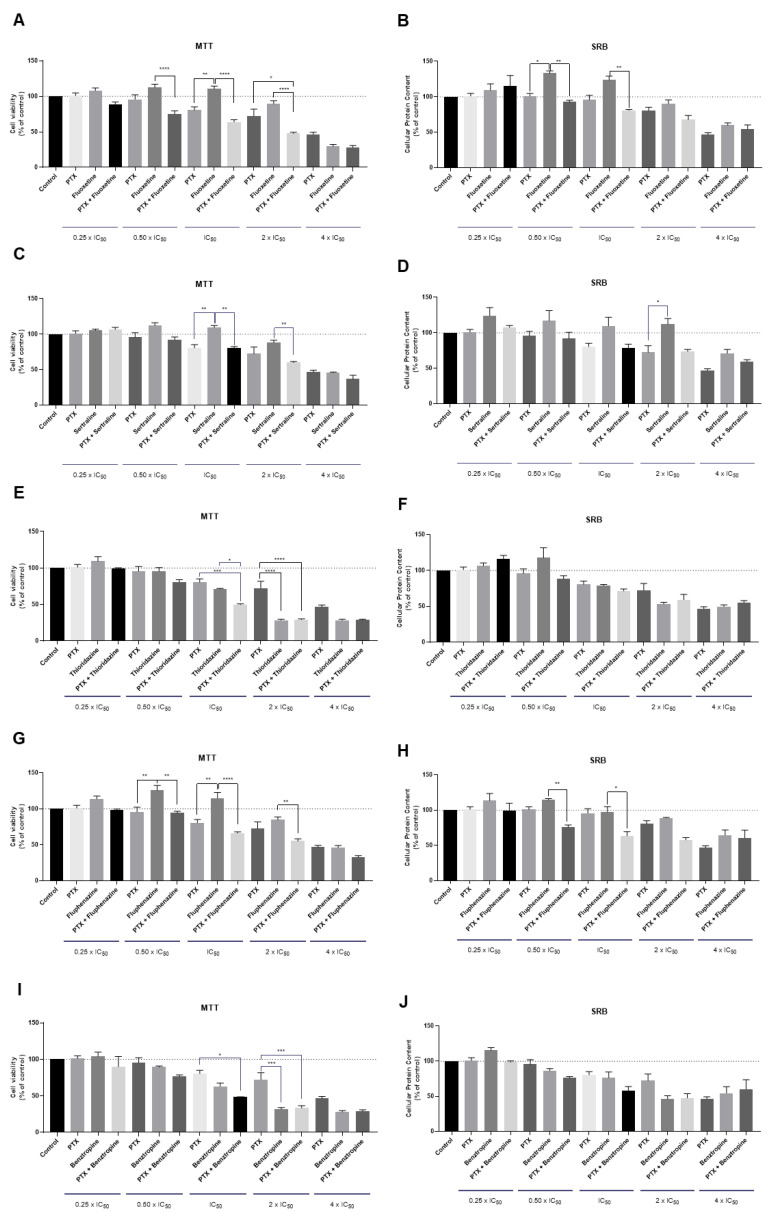
Growth inhibition of MCF-7 after 48 h of combination therapy with PTX, by MTT (**left**) and SRB assays (**right**). Cells were exposed to concentrations of each drug of 0.25, 0.5, 1, 2 and 4 times their IC_50_ and the cell viability was evaluated by MTT and SRB assays. The drugs in combination were co-administered at the same time. (**A**) The effect of PTX plus fluoxetine on cell viability and (**B**) cell protein synthesis. (**C**) The effect of PTX plus sertraline on cell viability and (**D**) cell protein synthesis. (**E**) The effect of PTX plus thioridazine on cell viability and (**F**) cell protein synthesis. (**G**) The effect of PTX plus fluphenazine on cell viability and (**H**) cell protein synthesis. (**I**) The effect of PTX plus benztropine on cell viability and (**J**) cell protein synthesis. Values are expressed in percentage of control and represent means ± SEM. Each experiment was done three times independently (*n* = 3); * statistically significant vs. control at *p* < 0.05. ** statistically significant vs. control at *p* < 0.01. *** statistically significant vs. control at *p* < 0.001. **** statistically significant vs. control at *p* < 0.0001.

**Figure 16:**
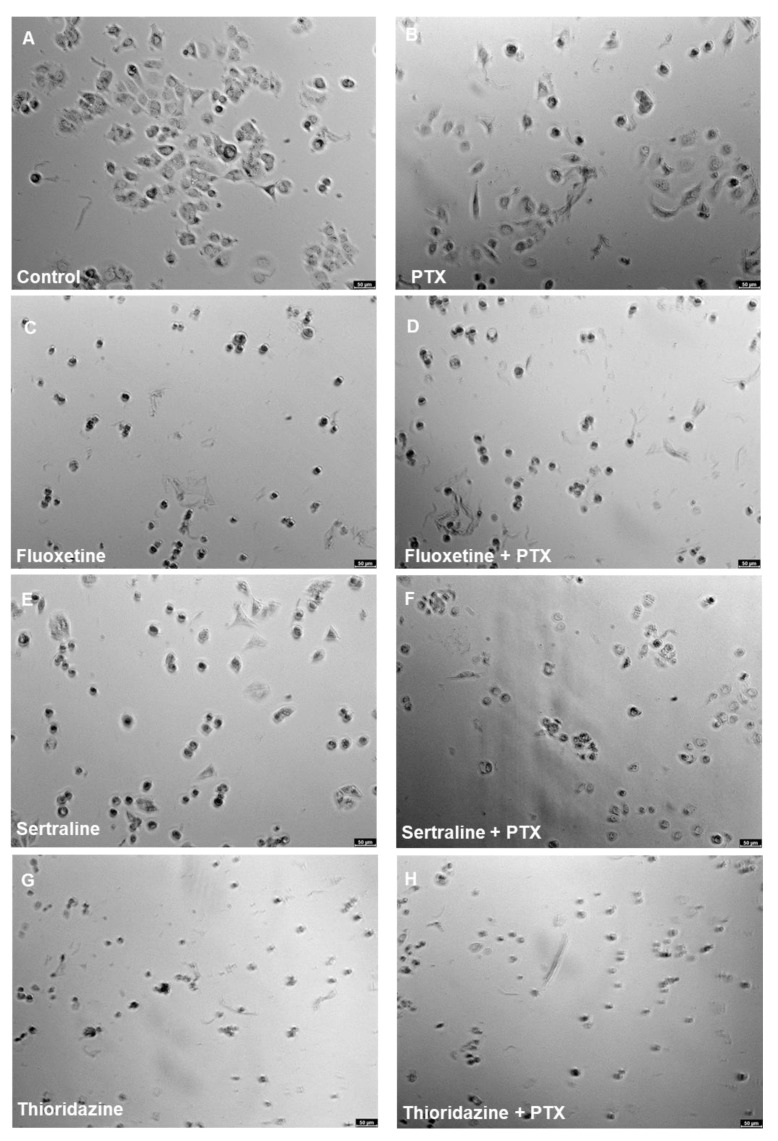

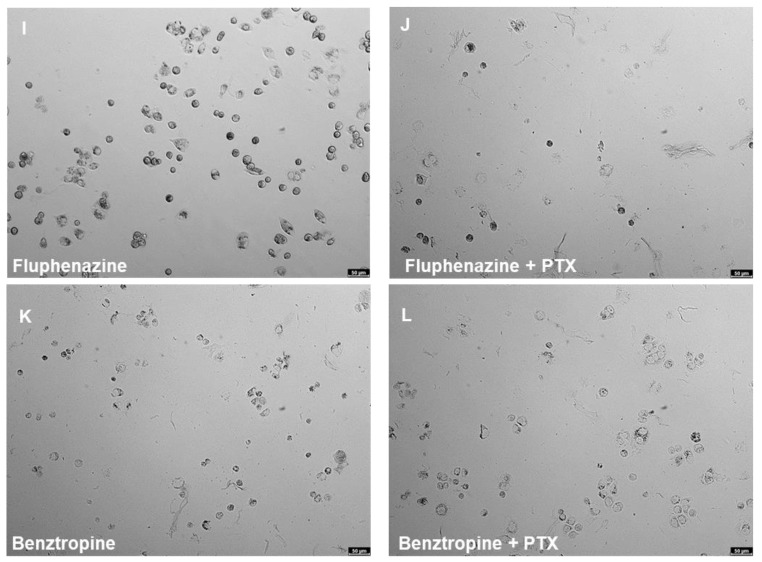
Microscopic cellular visualisation of MCF-7 cells after 48 h of incubation with vehicle (**A**), PTX (**B**), fluoxetine (**C**), fluoxetine + PTX (**D**), sertraline (**E**), sertraline + PTX (**F**), thioridazine (**G**), thioridazine + PTX (**H**), fluphenazine (**I**) fluphenazine + PTX (**J**), benztropine (**K**) and benztropine + PTX (**L**) at concentrations of 4 × IC_50_ of each drug. Scale bar: 50 µm.

We next evaluated the combination of PTX with each drug using the model of combination developed in our previous work [37]. The IC_50_ value for PTX adopted in these drug combinations was obtained in our previous work [37]. MCF-7 cells were treated with the two drugs alone or combined in a fixed ratio, in the concentrations of 0.25 × IC_50_, 0.5 × IC_50_, IC_50_, 2 × IC_50_ and 4 × IC_50_, and two cell-based assays were performed: MTT and SRB. Morphological evaluation of cells treated with each drug alone and in combination was also done. The combination of PTX plus fluphenazine, fluoxetine, benztropine, thioridazine and sertraline was evaluated. When combined with PTX, fluoxetine demonstrates significant anti-cancer effects both by MTT and SRB assays (Figure 15A,B, respectively), mainly at the concentration of 2 × IC_50,_ where the combined effect was statistically significant compared to each drug alone. The combination with sertraline resulted in a similar reduction of cell viability and cell protein synthesis as PTX alone, at all concentrations. At the concentrations of IC_50_ and 2 × IC_50_, the combination of PTX plus sertraline demonstrated significative anticancer effects compared to sertraline alone (Figure 15C,D). The combination with PTX and thioridazine demonstrated significant anticancer effects compared to both drugs alone, at concentrations of IC_50_ and 2 × IC_50_ (Figure 15E,F). The combination with fluphenazine resulted in all intermediate concentrations showing a greater anticancer effect than fluphenazine alone (Figure 15G,H). The activity seen for these combinations can be the result of the strong anticancer activity of PTX alone. The combination with benztropine resulted in a statistically significant reduction of cell viability at concentrations of IC_50_ and 2 × IC_50_ compared to PTX alone (Figure 15I,J), demonstrating that the activity of this combination can be the result of the repurposed drug alone, contrary to the previous combinations. Together, these results demonstrate that both CNS drugs and PTX can have different pharmacological actions in the combined effects. Morphologically, the results are in agreement with the MTT and SRB assays. At concentrations of 4 × IC_50_, all combinations resulted in a decrease of cell number and smaller and rounder cells, compared to control cells and PTX, which is indicative of cell death (Figure 16).

#### 2.2.3. Synergistic Combinations of PTX and CNS Agents

**Figure 17:**
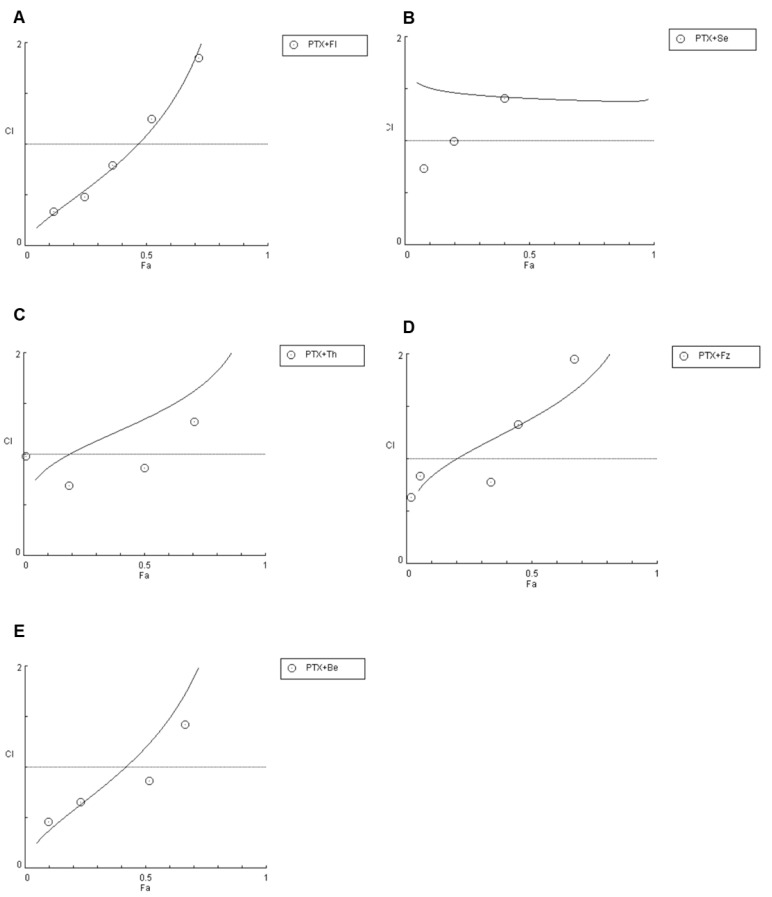
Chou-Talalay method Fa-CI plot of PTX plus fluoxetine (**A**), sertraline (**B**), thioridazine (**C**), fluphenazine (**D**) and benztropine (**E**). CI was plotted on the *y*-axis as a function of effect level (Fa) on the *x*-axis to evaluate drug synergism. CI < 1, CI = 1 and CI > 1 refers to synergism, additivity and antagonism, respectively.

Next, we calculated the combination index (CI) using the Chou-Talalay method, using the CompuSyn software. A combination of PTX plus fluoxetine demonstrated synergism for the lowest concentrations with three synergic pairs (Figure 17A), with Fa values of 0.1184, 0.2472 and 0.3621 (Table 6). The combination with sertraline resulted in only one synergic pair, for the lowest concentration (Figure 17B). The combinations of PTX plus thioridazine demonstrated synergism for two pairs (Figure 17C), with Fa values of 0.1895 and 0.5027 (Table 6). Combination with fluphenazine and benztropine resulted in three synergic pairs (Figure 17D,E, respectively), with Fa values lower than 0.60 (Table 6). Together, these results demonstrate that these CNS agents may be promising candidates to evaluate in future combinations.

**Figure 18:**
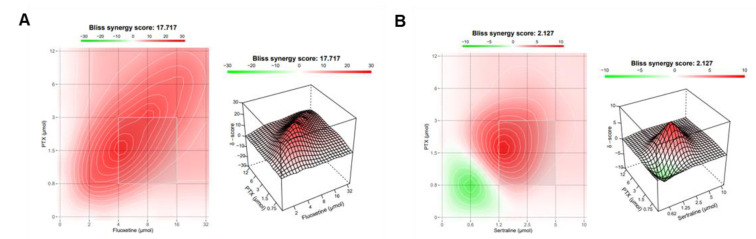

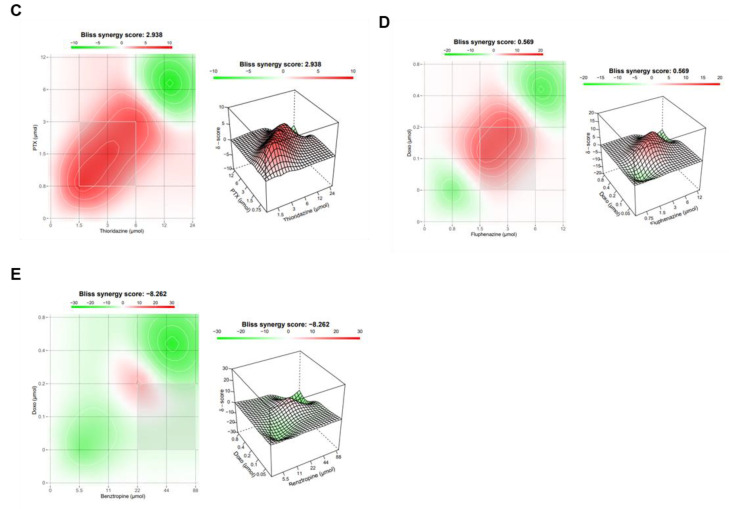
Bliss synergy plots of PTX plus fluoxetine (**A**), sertraline (**B**), thioridazine (**C**), fluphenazine (**D**) and benztropine (**E**).

These drug interactions were also evaluated by the Bliss Independence method, using the SynergyFinder 2.0 software. Fluoxetine combination with PTX analysed with the Bliss model (Figure 18A) demonstrated the highest synergy score, in line with the Chou-Talalay results, indicating synergism for three pairs. Sertraline combination demonstrated synergism using the Bliss model, with a positive synergy score of 2.127 (Figure 18B). The combination of PTX with thioridazine also resulted in synergism using the Bliss method (Figure 18C) with a synergy score of 2.938. The combinations of PTX plus fluphenazine and benztropine resulted in the lowest synergy scores using the Bliss method, with scores of 0.569 and -8.262 (Figure 18D,E, respectively).

The Bliss method results demonstrate slightly different results regarding the synergy evaluation of drug combinations compared to the Chou-Talalay results, especially regarding fluphenazine and benztropine combinations. Despite this, these reference models produce similar results most of the time.

## 3. Discussion

Drug repurposing and drug combination are strategies that have become more popular over the years, representing a faster and cheaper strategy to identify new potential candidates for cancer therapy. Repurposed drugs are already available on the market for other diseases and have pharmacokinetics, pharmacodynamics and toxicological profiles that are well established, facilitating their approval for novel indications. The combination of drugs allows decreasing the therapeutical dose, reducing the side effects of the drugs. The combination of antineoplastic drugs with other drug classes has been explored in several studies, but few studies report the CNS drugs benefits for cancer therapy, both alone and in combination. 5-FU is an essential agent in the treatment of CRC, but its use is limited by its short half-life, high cytotoxicity and low bioavailability, which limit its benefits. PTX is an antineoplastic drug commonly used for the treatment of breast cancer, but its maximum therapeutic dosage is limited by the appearance of drug resistance and its side effects. To overcome these problems, higher doses and long-term use of these antineoplastic drugs is necessary, which increases its side effects. Current research aims to decrease the chemotherapeutic drugs doses and exposure time. Recent studies have investigated new drugs that can synergise with 5-FU or PTX, but, to our knowledge, none have explored CNS drugs in combination with 5-FU or PTX for CRC or breast cancer therapy.

We studied the potential anticancer activity of different CNS drugs in HT-29 colon and MCF-7 breast cancer cells and evaluated the potential synergistic effects of this class of drugs with 5-FU or PTX, antineoplastic drugs used for CRC and breast cancer treatment, respectively. First, several CNS drugs were screened by MTT assay to treat HT-29 and MCF-7 cells to evaluate their potential as repurposed drugs. Besides CNS drugs, we also evaluated three antimalarial drugs (chloroquine, artesunate and mefloquine) in this study based on our previous results in MCF-7 cells, to evaluate if their anticancer behaviour was maintained in a different cell line (HT-29). After an evaluation using MTT, the IC_50_ for each drug was determined, and those with an IC_50_ under 20 µM were selected for combination with 5-FU or PTX, depending on the cell type. We employed our previously described combination model in which cells were treated with the concentrations of 0.25, 0.5, 1, 2 and 4 times the IC_50_ of each drug, alone and in combination, using MTT and SRB assays. We next evaluated synergism by three different methods: Chou-Talalay, Bliss (for HT-29 and MCF-7 cells) and HSA (only for HT-29 cells). The Chou-Talalay method is based on the median-effect equation, derived from the mass-action law principle. This unified theory encompasses the Michaelis–Menten, Hill, Henderson–Hasselbalch and Scatchard equations in biochemistry and biophysics and provides a quantitative definition for additive effect (CI = 1), synergism (CI < 1) and antagonism (CI > 1) in drug combinations [58]. The Bliss independence model adopts a stochastic process in which two drugs produce their effects independently, and the expected combination effect can be calculated based on the probability of independent events [57]. The HSA model is one of the simplest reference models for synergism evaluation and states that the expected combination effect is the maximum of the single drug responses at corresponding concentrations. The synergy score for a drug combination is averaged over all the dose combination measurements, giving a positive or negative value, corresponding to synergism or antagonism, respectively. The 2D and 3D synergy maps highlight synergistic and antagonistic dose regions in red and green colours, respectively.

Our results demonstrated that CNS drugs as single agents have the ability to decrease cell viability in a concentration-dependent manner in both cell lines. In HT-29 colon cancer cells, the most promising drugs were latrepirdine, fluphenazine, fluoxetine, benztropine, thioridazine, sertraline, mefloquine and artesunate, all with IC_50_ values under 20 µM, with fluphenazine being the most potent with an IC_50_ of 1.86 µM. For MCF-7 breast cancer cells, we found out that these drugs were less potent, with IC_50_ values higher than the ones obtained for colon cancer cells, except for sertraline, whose IC_50_ was 2.22 µM.

In simultaneous combination, we found that sertraline and thioridazine were the most promising candidates for the improvement of the anti-cancer activity of 5-FU in HT-29 colon cancer cells. For MCF-7 cells, almost all tested combinations resulted in synergic pairs, for the lowest concentrations. Drugs such as fluoxetine and thioridazine combined with PTX resulted in an enhanced reduction in the viability of MCF-7 cells, compared to a single treatment with repurposed drugs or PTX. Compared to HT-29 cells, the combination of CNS drugs with PTX in MCF-7 revealed more synergistic interactions than with 5-FU, except for sertraline. Curiously, when tested alone in MCF-7, sertraline was the most potent repurposed drug, but its combination with PTX resulted in only one synergistic pair. Altogether, these results suggest that the dominant behaviour of drug combinations comes from the antineoplastic drug and that the mechanism of these drug combinations may be related to this class of drugs.

Specifically for fluphenazine, fluoxetine, benztropine and artesunate in HT-29 cells, we found out that the combination of these drugs with 5-FU resulted in worse results than the repurposed drug alone, probably due to competition between the two drugs, so we designed a model of drug combination based on the sequential addition of the two drugs, with an interval of 24 h. For most drugs, we did not find significant differences between the drug schedules, except for artesunate, in which we found that artesunate prior to 5-FU administration resulted in enhanced anticancer effects. For fluphenazine, fluoxetine and benztropine, we found that these drugs act better alone than in combination, being ideal candidates for drug repurposing. These results demonstrate for the first time that CNS agents may be potential candidates for drug repurposing in colon and breast cancer therapy. We have found that all tested CNS drugs can synergistically decrease MCF-7 cell viability when combined with PTX, with fluoxetine, benztropine and fluphenazine being the most promising drugs at lower concentrations. We also concluded that sertraline and thioridazine combination with 5-FU can synergistically decrease cancer viability in HT-29 colon cancer cells. We prove that artesunate, an antimalarial drug, has anticancer potential in these cells and that the combination with 5-FU is beneficial if given in a sequential schedule and prior to 5-FU.

Mechanistically, several studies suggest that combination treatment with 5-FU synergistically induces apoptosis in colon cancer cells [59,60,61,62,63,64]. Despite inducing apoptosis, the observed synergistic effect can also be the result of the combined impact on autophagy, a catabolic process exerted in cells in response to stressful conditions, such as nutrient deprivation or damage to proteins/DNA, which can ultimately trigger cell death. Indeed, in human colon cancer cell lines and colorectal cancer-xenografted mice, sertraline demonstrated proapoptotic activity by mitogen-activated protein kinase cascade activation and *Bcl-2* inhibition [65]. Regarding thioridazine, recently, it was found that this drug significantly suppresses the proliferation and invasion of colon cancer stem cells and induced cell apoptosis in a concentration-dependent manner. It was found that apoptosis genes such as *Bax* and *caspase-3* are overexpressed after treatment, and anti-apoptosis gene *Bcl-2* was downregulated. Accordingly, the mitochondrial potential of these cells was downregulated [66]. Based on these literature findings, we propose that simultaneous apoptosis and autophagic cell death can be occurring in our combinations. We believe that 5-FU combined with sertraline and thioridazine mainly increases the concentration of caspase-3 enzyme and other apoptotic proteins in HT-29 cells causing apoptosis-dependent cell death. This inhibition of autophagy and induction of apoptosis can be proposed to be the basis of synergy in the case of the combined treatment of 5-FU and these CNS drugs in colon cells.

Regarding the MCF-7 results, fluoxetine, fluphenazine and benztropine combined with PTX revealed to be the most promising combinations. PTX belongs to the taxanes drug class and acts by blocking cell mitosis through the stabilisation of microtubules, leading to cell cycle arrest preferentially in the G2/M phase and apoptosis [67]. Some studies showed that drugs acting on serotonin (5-HT) signalling, including selective serotonin reuptake inhibitors (SSRIs), inhibit tumour sphere formation in human breast tumour cells in in vitro and in vivo models [68]. Particularly, fluoxetine was found to significantly decrease the proliferation of several breast cancer cell lines by inducing apoptosis and autophagy-mediated cell death or endoplasmic reticulum stress and autophagy, respectively [68,69,70,71]. In triple-negative breast cancer cells, fluphenazine inhibited breast cancer cell growth and induced G0/G1 cell cycle arrest and induced mitochondria-mediated apoptosis in breast cancer cells [72]. In the case of MCF-7 cells, as they do not express caspase-3, they do not undergo normal apoptosis and autophagy can represent the main alternative cell death pathway [73]. Recent studies suggest that benztropine reduces the activity of oncogenic signalling transducers and trans-activators for MMP9, including STAT3, NF-*κ*B and β-catenin [74]. We believe that the PTX mechanism of synergy in combination with CNS drugs can be related to enhanced cell cycle arrest, interference with important oncogenic signalling and increased autophagy-mediated cell death. We also believe that CNS drugs can act as chemosensitizers by slowing down drug efflux, increasing drug accumulation. As several CNS drugs are substrates and modulators of the P-glycoprotein (P-gp) protein [75,76,77], we also believe they can inhibit P-gp to stop effusing drugs from intracellular, to increase the intracellular concentration of anticancer drugs such as PTX.

These results imply that CNS drugs may be promising chemosensitizers compounds and enhance the cytotoxic effects of 5-FU and PTX in HT-29 and MCF-7 cancer cells, respectively. Since these drugs are already accessible in the market, their use for cancer therapy is achievable. Since different colon cancer cells lines are metabolically different and have specific characteristics, more research should be made on other colon cancer cells, such as HCT116, SW480, LoVo, etc. The same is true for breast cancer cells, and these combinations can be further explored in other cell lines such as tumoral MDA-MB-231 cells or normal MCF-10A cells. Deeper mechanistic studies are strongly recommended to evaluate the anticancer mechanisms underlying these drugs and these combinations. This class of drugs should also be investigated both alone and in combination for other types of cancer, such as pancreatic, prostate, lung, etc. These are promising results and should be further confirmed in animal models and clinical trials. Our results demonstrate that the use of CNS and antimalarial drugs, both alone and in combination, may lead to new therapeutic strategies for colon and breast cancer therapy.

## 4. Materials and Methods

### 4.1. Materials

McCoy’s 5A Modified Medium, Dulbecco’s Modified Eagle Medium (DMEM), foetal bovine serum (FBS) and a penicillin-streptomycin solution were purchased from Millipore Sigma (Merck KGaA, Darmstadt, Germany). Other cell culture reagents were purchased from Gibco (Thermo Fisher Scientific, Inc., Waltham, MA, USA). 5-FU (cat. no. F6627), selegiline (cat. no. M003), entacapone (cat. no. SML0654), tolcapone (cat. no. SML0150), latrepirdine (cat. no. D6196), fluphenazine (cat. no. F4765), safinamide (cat. no. SML0025), carbidopa (cat. no. PHR1655), scopolamine (cat. no. S1013), Thiazolyl Blue Tetrazolium Bromide (MTT, cat. no. M5655) and sulforhodamine B (SRB, cat. no. S1402) were obtained from Sigma-Aldrich (Merck KGaA, Darmstadt, Germany). Benztropine (cat. no. 16214), thioridazine (cat. no. 14400), fluoxetine (cat. no. 14418) and artesunate (cat. no. 11817) were obtained from Cayman Chemical (Ann Arbor, MI, USA). Nepicastat (cat. no. 5037) and paclitaxel (cat. no. 1097) were obtained from Tocris Bioscience (Bristol, UK). Mefloquine (cat. no. sc-211784) and chloroquine (cat. no. C6628) were purchased from Santa Cruz Biotechnology (Dallas, TX, USA). Bromocriptine was used in tablets and diluted in water before stock preparation.

### 4.2. Cell Line and Cell Culture

Human colorectal cancer HT-29 and breast cancer MCF-7 cell lines were obtained from the American Type Culture Collection (ATCC; Manassas, VA, USA) and maintained according to ATCC’s recommendations at 37 °C and 5% CO_2_ in appropriate medium supplemented with 10% foetal bovine serum, 100 U/mL penicillin G and 100 µg/mL streptomycin. Cells were maintained in the logarithmic growth phase at all times. The media was changed every 2 days and trypsinised with 0.25% trypsin-EDTA. A total of 200 µL of HT-29 cells (7500 cells/well) or MCF-7 cells (5000 cells/well) were seeded in 96-well plates and allowed to adhere overnight before drug exposure. After 24 h, the cell culture media were replaced with 200 µL of drug-containing media. Cells were exposed to drugs for 48 h, followed by MTT and SRB assays to evaluate single and combination drug treatments in the cell viability and protein synthesis rate of these cells.

### 4.3. Drug Treatment

The half-maximal inhibitory concentration (IC_50_) value was first determined for each drug alone in HT-29 and MCF-7cells. Drug concentrations ranged from 0.1 to 100 µM for the single-drug treatment. Combination studies were performed by combining 5-FU or PTX (Drug A) according to each cell line, with different repurposed drugs (Drug B). Drug A was 5-FU for HT-29 cells and PTX for MCF-7 cells. Only drugs that present the most promising pharmacological profile (IC_50_ < 20 µM) were tested in simultaneous combination with 5-FU or PTX, following schedule A (Figure 11). Both Drug A and Drug B concentrations were variable, and the combined effects of equipotent concentrations (fixed ratio) of the IC_50_ values for each drug were evaluated. The combinations of fluoxetine, fluphenazine, benztropine and artesunate with 5-FU were also tested in sequential schedules of administration (Schedule B and C, Figure 11). For schedule A, cells were treated concomitantly with 5-FU or PTX and each repurposed drug for 48 h. For schedule B, cells were pre-treated with 5-FU 24 h followed by each repurposed drug for 24 h. For schedule C, cells were pre-treated with each repurposed drug for 24 h followed by 5-FU for another 24 h.

### 4.4. Cell Viability Assay

To determine the effects of 5-FU or PTX and the repurposed drugs on the viability of HT-29 and MCF-7 cells, respectively, MTT and SRB assays were used. For the MTT protocol, after drug treatment, the cell medium was removed and 100 µL/well of MTT solution (0.5 mg/mL in PBS) was added. Cells were incubated for 3 h, protected from light. After this period, the MTT solution was removed, and DMSO (100 µL/well) was added to solubilise the formazan crystals. Absorbance was measured at 570 nm in an automated microplate reader (Tecan Infinite M200, Tecan Group Ltd., Männedorf, Switzerland). For SRB assay, after treatments, the cultured cells were fixed with ice-cold 10% trichloroacetic acid for 30 min and stained with 0.4% SRB for 1 h at room temperature. Excess dye was removed by rinsing several times with tap water. Protein-bound dye was dissolved with 200 µL 10 mM Tris base solution for the determination of absorbance with a microplate reader with a filter wavelength of 540 nm (Tecan Infinite M200, Tecan Group Ltd., Männedorf, Switzerland). The IC_50_ of the therapeutic drug was determined as each drug concentration showing 50% cell growth inhibition as compared with control. All conditions were performed three times independently, in triplicate.

### 4.5. Cell Morphology Visualisation

After each treatment, cell morphology was assessed on a Leica DMI 6000B microscope equipped with a Leica DFC350 FX camera and then analysed with the Leica LAS X imaging software (v3.7.4).

### 4.6. Data Analysis

GraphPad Prism 8 (GraphPad Software Inc., San Diego, CA, USA) was used to produce concentration-response curves by nonlinear regression analysis. The viability of cells treated with each drug was normalised to the viability of control cells and cell viability fractions were plotted vs. drug concentrations in the logarithmic scale.

### 4.7. Analysis of Drug Interactions

To quantify drug interaction between 5-FU and CNS drugs, we first estimated the Combination Index (CI) by the unified theory, introduced by Chou and Talalay [15] using the CompuSyn software (ComboSyn, Inc., New York, NY, USA). We used the mutually exclusive model, based on the assumption that drugs act through entirely different mechanisms [78]. The two drugs were combined in a fixed ratio of doses that correspond to 0.25, 0.5, 1, 2 and 4 times that of the individual IC_50_ values. CI was plotted on the *y*-axis as a function of effect level (Fa) on the *x*-axis to assess drug synergism between drug combinations. The CI is a quantitative representation of pharmacological interactions. CI < 1 indicates synergism, CI = 1 indicates additive interaction and CI > 1 indicates antagonism. We also estimated the expected drug combination responses based on the highest single agent (HSA) and Bliss reference model using SynergyFinder [57]. Deviations between observed and expected responses with positive and negative values denote synergy and antagonism, respectively.

### 4.8. Statistical Analysis

The results are presented as mean ± SEM for n experiments performed. All data were assayed in three independent experiences, in triplicate. Statistical comparisons between control and treatment groups, at the same time point, were performed with Student’s *t*-test and one-way ANOVA test. Statistical significance was accepted at *p* values < 0.05.

## Figures and Tables

**Table 1:** CNS drugs used in this work and their mechanism of action.

Drug	Mechanism of Action	Ref.
Selegiline	Irreversible inhibitor of monoamine oxidase type B (MAO-B). Binds to MAO-B and blocks the microsomal metabolism of dopamine, enhancing the dopaminergic activity in the substantial nigra. It can also inhibit monoamine oxidase type A (MAO-A).	[39]
Safinamide	Reversible inhibitor of MAO-B; blocks voltage-dependent Na^+^ and Ca^2+^ channels and inhibits the glutamate release.	[40]
Entacapone	Adjunct to levodopa/carbidopa therapy and reversible inhibitor of catechol-O-methyltransferase (COMT) in peripheral tissues, altering the plasma pharmacokinetics of levodopa.	
Tolcapone	[41,42]
Latrepirdine	Blocks H1 histamine receptor activity and interact with calcium channels and a wide range of other neurotransmitter receptors.	[43]
Fluphenazine	Blocks postsynaptic mesolimbic dopaminergic D1 and D2 receptors in the brain and depresses the release of hypothalamic and hypophyseal hormones	
Thioridazine	[44,45]
Fluoxetine	Selective serotonin reuptake inhibitor (SSRI); inhibits the presynaptic reuptake of the neurotransmitter serotonin, increasing the levels of 5-hydroxytryptamine (5-HT) in the brain.	[46]
Benztropine	Selective inhibitor of dopamine transporter; presents affinity for histamine and M1 muscarine receptors.	[47]
Carbidopa	Inhibitor of the aromatic amino acid decarboxylase (DDC) and the peripheral metabolism of levodopa.	[48]
Bromocriptine	Agonist of dopaminergic D2 and D3 receptors in the brain.	[49]
Nepicastat	Inhibitor of Dopamine β-hydroxylase (DβH)	[50]
Scopolamine	Non-selective competitive inhibitor of G-protein-coupled muscarinic receptors (mAChRs), with anticholinergic action.	[51]
Carbamazepine	Inhibits sodium channel firing, treating seizure activity. In bipolar disorder, carbamazepine is thought to increase dopamine turnover and increase GABA transmission.	[52]
Sertraline	Selective inhibitor of serotonin reuptake at the presynaptic neuronal membrane, thereby increasing serotonergic activity.	[53]
Rivastigmine	Binds reversibly with and inactivates cholinesterase (e.g., acetylcholinesterase and butyrylcholinesterase), preventing the hydrolysis of acetylcholine, and thus, leading to an increased concentration of acetylcholine at cholinergic synapses.	[54]
*m*-chlorophenilbiguanide	Allosteric agonist and modulator of the 5-HT_3_ receptor and an antagonist of the α_2A_-adrenergic receptor.	[55,56]

**Table 2:** Cytotoxicity of 5-FU, several CNS drugs and some antimalarials agents in HT-29 colon cancer cells. IC_50_ values are given as mean.

Drug	IC_50_ (µM)
5-Fluorouracil	3.79 ^‡^
Selegiline	>100
Entacapone	40.89
Tolcapone	35.47
Latrepirdine	7.75 ^‡^
Fluphenazine	1.86 ^‡^
Safinamide	>100
Fluoxetine	6.12 ^‡^
Benztropine	18.23 ^‡^
Thioridazine	4.26 ^‡^
Carbidopa	>100
Bromocriptine	>100
Nepicastat	61.24
Scopolamine	>100
Carbamazepine	>100
Sertraline	2.45 ^‡^
Chloroquine	32.13
Mefloquine	11.49 ^‡^
Artesunate	17.88 ^‡^
Rivastigmine	>100
m-Chlorophenilbiguanide	>100

^‡^ Drugs selected for combinations with 5-FU.

**Table 3:** CI values and the respective fractional effect of different combinations of 5-FU plus CNS agents and antimalarial drugs. CI in red indicates concentrations of drug pairs that are synergic. Cells were treated with 0.25, 0.5, 1, 2 and 4 times the IC_50_ of each drug (total dose).

Combination (Drug A + Drug B)	Total Dose (Dose A + Dose B)	Fractional Effect (Fa)	CI Value
5-FU + latrepirdine	2.75	0.14129	1.65359
	5.5	0.16103	2.00449
	11.0	0.17389	2.97604
	22.0	0.21248	2.69396
	44.0	0.44126	0.21700
5-FU + fluoxetine	2.25	0.1497	1.83049
	4.5	0.1688	2.64547
	9.0	0.364	1.36014
	18.0	0.73678	0.98008
	36.0	0.8799	1.09726
5-FU + benztropine	5.25	0.1984	1.94601
	10.5	0.3913	1.17167
	21.0	0.5293	1.27070
	42.0	0.6237	1.67649
	84.0	0.8716	0.74841
5-FU + thioridazine	1.75	0.00001	3.28032
	3.5	0.0882	0.57237
	7.0	0.5245	0.63129
	14.0	0.7502	0.99126
	28.0	0.8692	1.63800
5-FU + sertraline	1.375	0.0001	0.91640
	2.75	0.001	0.95924
	5.5	0.1469	0.54137
	11.0	0.4412	0.76907
	22.0	0.8507	0.99970
5-FU + mefloquine	3.625	0.2138	29.9325
	7.25	0.5659	4.07663
	14.5	0.8481	0.68334
	29.0	0.861	1.14685
	58.0	0.873	1.92463
5-FU + artesunate	5.25	0.00001	1461.26
	10.5	0.0782	1.27561
	21.0	0.1521	1.35742
	42.0	0.2489	1.62381
	84.0	0.5575	1.07643
5-FU + fluphenazine	5.25	0.11608	11.6103
	10.5	0.17321	13.0845
	21.0	0.4477	8.40182
	42.0	0.5522	12.1699
	84.0	0.8399	8.04387

**Table 4:** CI values and respective fractional effect for three different combination schedules of 5-FU plus fluphenazine, fluoxetine, benztropine and artesunate. CI in red indicates concentrations of drug pairs that are synergic.

Combination Indexes for the Different Drug Combinations				
Drug Combination (µM)		Schedule A (CI)	Schedule B (CI)	Schedule C (CI)
*5-FU*	*Fluphenazine*			
0.75	0.5	11.61	>100	4.09
1.5	1	13.08	>100	24.81
3	2	8.40	>100	1.77
6	4	12.17	4.36	1.96
12	8	8.04	4.68	1.76
*5-FU*	*Fluoxetine*			
0.75	1.5	1.83	>100	7.30
1.5	3	2.65	>100	3.53
3	6	1.36	>100	2.32
6	12	0.98	2.56	1.48
12	24	1.10	1.74	1.11
*5-FU*	*Benztropine*			
0.75	4.5	1.95	34.97	4.01
1.5	9	1.17	2.86	2.54
3	18	1.27	3.42	2.12
6	36	1.68	4.33	3.58
12	72	0.75	4.31	3.14
*5-FU*	*Artesunate*			
0.75	4.5	>100	7.88	0.40
1.5	9	1.28	4.85	0.49
3	18	1.36	4.22	0.81
6	36	1.62	2.56	1.16
12	72	1.08	1.64	1.26

**Table 5:** Comparison between the IC_50_ of several CNS drugs in HT-29 and MCF-7 cancer cells. IC_50_ values are given as mean.

Drug	HT-29 IC_50_ (µM)	MCF-7 IC_50_ (µM)
Fluphenazine	1.86	2.68 ^‡^
Fluoxetine	6.12	7.78 ^‡^
Benztropine	18.23	21.71 ^‡^
Thioridazine	4.26	5.72 ^‡^
Sertraline	2.45	2.22 ^‡^
Latrepirdine	7.75	75.37

^‡^ Drugs selected for combinations with PTX.

**Table 6:** CI values and respective fractional effect of different combinations of PTX plus CNS agents. CI in red indicates concentrations of drug pairs that are synergic. Cells were treated with 0.25, 0.5, 1, 2 and 4 times the IC_50_ of each drug (total dose).

Combination (Drug A + Drug B)	Total Dose (Dose A + Dose B)	Fractional Effect (Fa)	CI Value
PTX + fluoxetine	2.75	0.1184	0.33562
	5.5	0.2472	0.48322
	11.0	0.3621	0.79198
	22.0	0.5227	1.24993
	44.0	0.7167	1.85505
PTX + sertraline	1.375	1.0 × 10^−4^	5.01852
	2.75	0.0768	0.73865
	5.5	0.1987	0.99737
	11.0	0.4012	1.40860
	22.0	0.6326	2.04261
PTX + thioridazine	2.25	0.0109	0.98541
	4.5	0.1895	0.69489
	9.0	0.5027	0.86789
	18.0	0.7077	1.32210
	36.0	0.7128	2.62409
PTX + fluphenazine	1.5	0.01908	0.63552
	3.0	0.057	0.83495
	6.0	0.33804	0.78096
	12.0	0.44769	1.33256
	24.0	0.67095	1.95345
PTX + benztropine	6.25	0.09897	0.45763
	12.5	0.2321	0.65346
	25.0	0.51629	0.86840
	50.0	0.66485	1.42716
	100.0	0.71074	2.66897

## Data Availability

Not applicable.
